# Comprehensive proteome analysis of nasal lavage samples after controlled exposure to welding nanoparticles shows an induced acute phase and a nuclear receptor, LXR/RXR, activation that influence the status of the extracellular matrix

**DOI:** 10.1186/s12014-018-9196-y

**Published:** 2018-05-11

**Authors:** Neserin Ali, Stefan Ljunggren, Helen M. Karlsson, Aneta Wierzbicka, Joakim Pagels, Christina Isaxon, Anders Gudmundsson, Jenny Rissler, Jörn Nielsen, Christian H. Lindh, Monica Kåredal

**Affiliations:** 1 Division of Occupational and Environmental Medicine, Lund University, Lund, Sweden; 2 Occupational and Environmental Medicine Center, Department of Clinical and Experimental Medicine, Linköping University, Linköping, Sweden; 3 Department of Design Sciences, Ergonomics and Aerosol Technology, Lund University, Lund, Sweden

**Keywords:** Chamber study, Welding fume particles, Nasal lavage, Effects, Proteomics, Mass spectrometry, Pathways

## Abstract

**Background:**

Epidemiological studies have shown that many welders experience respiratory symptoms. During the welding process a large number of airborne nanosized particles are generated, which might be inhaled and deposited in the respiratory tract. Knowledge of the underlying mechanisms behind observed symptoms is still partly lacking, although inflammation is suggested to play a central role. The aim of this study was to investigate the effects of welding fume particle exposure on the proteome expression level in welders suffering from respiratory symptoms, and changes in protein mediators in nasal lavage samples were analyzed. Such mediators will be helpful to clarify the pathomechanisms behind welding fume particle-induced effects.

**Methods:**

In an exposure chamber, 11 welders with work-related symptoms in the lower airways during the last month were exposed to mild-steel welding fume particles (1 mg/m^3^) and to filtered air, respectively, in a double-blind manner. Nasal lavage samples were collected before, immediately after, and the day after exposure. The proteins in the nasal lavage were analyzed with two different mass spectrometry approaches, label-free discovery shotgun LC–MS/MS and a targeted selected reaction monitoring LC–MS/MS analyzing 130 proteins and four in vivo peptide degradation products.

**Results:**

The analysis revealed 30 significantly changed proteins that were associated with two main pathways; activation of acute phase response signaling and activation of LXR/RXR, which is a nuclear receptor family involved in lipid signaling. Connective tissue proteins and proteins controlling the degradation of such tissues, including two different matrix metalloprotease proteins, MMP8 and MMP9, were among the significantly changed enzymes and were identified as important key players in the pathways.

**Conclusion:**

Exposure to mild-steel welding fume particles causes measurable changes on the proteome level in nasal lavage matrix in exposed welders, although no clinical symptoms were manifested. The results suggested that the exposure causes an immediate effect on the proteome level involving acute phase proteins and mediators regulating lipid signaling. Proteases involved in maintaining the balance between the formation and degradation of extracellular matrix proteins are important key proteins in the induced effects.

**Electronic supplementary material:**

The online version of this article (10.1186/s12014-018-9196-y) contains supplementary material, which is available to authorized users.

## Background

Occupations with work tasks that result in high emissions of airborne particles pose a risk for respiratory health effects according to the national institution of occupational safety and health, occupational safety and health administration, and the world health organization [1–4]. Welding exposure has been shown to induce a number of negative health effects and symptoms, including bronchitis, siderosis, asthma, metal fume fever, lung function changes, immune suppression, pulmonary infection, and cancer [5, 6]. During welding, base materials and a filler material are fused at high temperature using different methods, e.g. gas metal arc welding and manual metal arc welding and a mixture of gases and agglomerated particles (networks of interconnected particles) are released into the air [7, 8]. The sizes of the agglomerates (typically ~ 100–1000 nm) are in the respirable range, which means that they can be inhaled and exert potentially negative airway effects. The agglomerates are typically built up of primary nanoparticles, which are in the range of 2–70 nm in diameter [9]. The welding fume particles consist of a complex mixture of different metals, e.g. iron, manganese metal oxides [10], and other trace elements depending on the type of welding method and the electrode material, e.g. mild steel or stainless steel [8].

It has been shown that although the exposure levels do not normally exceed current Swedish permissible occupational exposure limits for inorganic respiratory dust (5 mg/m^3^), there is a high frequency of upper and lower respiratory symptoms among welders [11, 12]. Previous studies have suggested that welding particle exposure induces oxidative stress and an inflammatory response [13, 14]. Elevated levels of tumor necrosis factor-α (TNF-α), interleukin (IL)-1, IL-6, and IL-8 have been observed after welding exposure [15–17]. It has also been shown that even at low exposure levels in a controlled human chamber study where no clinical symptoms were recorded, biomarkers of inflammation were still induced [18]. There is a need for a deeper understanding of the mechanisms involved in the responses induced by welding fume particle exposure, and the identification of proteins and pathways associated with welding fume particle exposure is an important step in this regard.

A combination of different mass spectrometry techniques can rapidly and comprehensively identify and quantify protein contents in highly complex samples [19]. During the discovery phase, shotgun proteomics can serve as a valuable tool in rapidly generating a global profile of the protein changes linked to a specific event. Processing the results from the discovery analysis with the bioinformatics-based *ingenuity pathway* analysis (IPA) can generate hypotheses on the mechanistic pathways that are induced [20]. Measurement of the identified proteins with a targeted LC-coupled selected reaction monitoring (LC–SRM) method offers a better opportunity to validate multiple biomarker candidates simultaneously because it offers high-throughput analysis and is able to provide label-free relative quantification as well as absolute protein quantification.

The aim of this study was to investigate the effects on the proteome level induced by welding fume particles at exposure levels well below the present Swedish permissible occupational exposure limit in welders with work-related lower airway symptoms.

## Methods

### Materials

Amicon Ultra-0.5 centrifugal filters (cut-off 3 kDa) were purchased from Millipore (Bedford, MA, USA). The micro bicinchoninic acid (BCA) protein assay kit was from Thermo Scientific (Rockford, IL, USA). Trypsin (sequencing grade) was purchased from Promega Corporation (Madison, WI, USA). Calcium chloride, formic acid (FA), hydrochloric acid, and ammonium acetate were purchased from Merck (Darmstadt, Germany). Dithiothreitol (DTT) and iodoacetamide were purchased from Sigma-Aldrich (St. Louis, MO, USA). Acetonitrile (ACN) was purchased from Lab-scan (Dublin, Ireland).

### Study subjects

Welders (n = 11) with work-related lower airway symptoms (wheezing, dyspnoea, and/or cough) in the last month were recruited for the study. The inclusion criteria are described in more detail by Dierschke et al. [13]. Briefly, the study subjects were all non-smoking men ranging from 29 to 66 years of age. Baseline medical examinations of the participants were conducted and included standardized questions about past and current respiratory and cardiovascular diseases and allergies. Work-related symptoms (defined as symptoms that recovered during weekends and holidays) from the upper airway at least once a week during the last month were also recorded. Six welders reported work-related nasal symptoms (runny nose, itching/sneezing) during the last month, and three welders showed a positive skin prick test for a standard panel of aeroallergens. Furthermore, lung function (FVC and FEV_1_ as % of predicted) and methacholine test (PD_15_) were carried out [18]. For the whole group the lung function was normal: FVC: 99 (72–114) and FEV_1_: 101 (66–132; % of predicted, median, min–max). Two out of 10 subjects had signs of bronchial hyperreactivity (PD_15_ < 167 µg methacholine). One welder started coughing during the test, thus it could not be completed. The two hyperreactive welders were among the three subjects with slightly decreased lung function.

### Study design

**Fig. 1:**
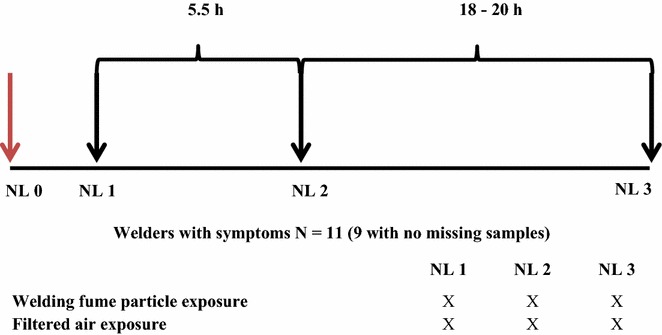
Overview of study design and nasal lavage sampling. The subjects were exposed for 5.5 h to either filtered air or welding fumes at 1 mg/m^3^ (PM_2.5_). Nasal lavage fluids were collected on four occasions, twice prior to exposure and twice after. The first sample was a washout (NL 0, not analyzed), the second was the baseline sample (NL 1), which was taken 30 min after the wash out, the third sample was collected immediately at the end of exposure (NL 2), and the fourth sample was taken 18–20 h after the end of the exposure (NL 3). Two subjects had missing samples and were therefore excluded in the analysis of the pooled samples

The subjects were exposed in an exposure chamber (22 m^3^) for 5.5 h with a lunch break for 1 h after 2.5 h of exposure to either filtered air or welding fumes at a concentration of 1 mg/m^3^ (PM_2,5_) as described previously [21]. Briefly, particles were generated by gas-metal arc welding in mild steel and collected in a closed chamber from where they were diluted in fresh air via a carbon filter for removal of gases and then fed into the exposure chamber. The composition of the welding fume particles was mainly iron oxides and up to 20% manganese. The primary particle size ranged from 2 to 70 nm, and aggregates with a mean mobility diameter of 160 nm were formed. The exposures were conducted in a double blind fashion and were performed on two Mondays with 1 week in between, making sure that no occupational exposure to welding occurred for at least 48 h prior to the experimental exposure. Nasal lavage fluids were collected on four occasions, twice prior to exposure and twice after, by instilling the nasal cavity with 18 mL of an isotonic saline solution. The first sample was a washout (NL 0, not analyzed), and the second was the baseline sample (NL 1) that was taken 30 min after the wash out. The third sample was collected immediately at the end of the exposure (NL 2), and the fourth was taken at 18–20 h after the end of the exposure (NL 3). An overview of the exposure and sample collection is given in Fig. 1. All samples were stored at − 80 °C until analysis. Two subjects had missing samples and were therefore excluded in the analysis of the pooled samples.

### Samples

**Fig. 2:**
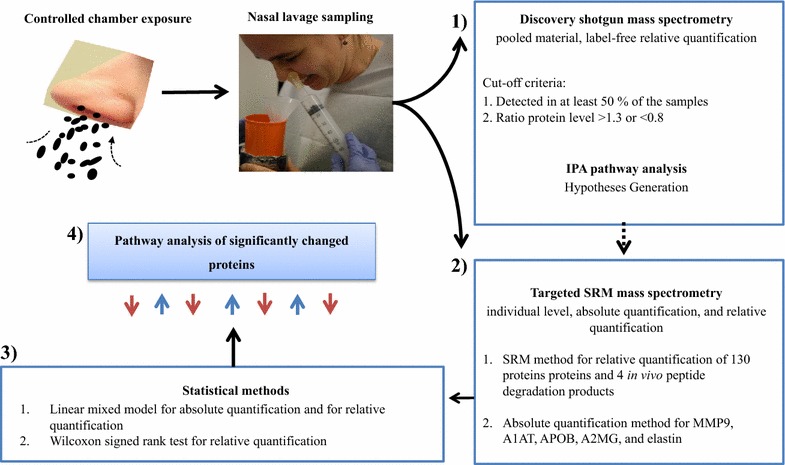
Flowchart of the experiment: Nasal lavage sampling before and after exposure to welding fume particles and filtered air. (1) Protein analysis through shotgun proteomics in combination with IPA analysis generates a hypothesis of the mechanisms induced by the welding fume particle exposure. (2) Targeted protein analysis of specific proteins with label-free relative quantification and absolute quantification of MMP9, A1AT, A2MG, APOB, and elastin. (3) Statistical evaluation of the changed protein level with linear mixed model and Wilcoxon signed rank test. (4) Pathway analysis of significantly changed proteins after welding fume particle exposure compared to filtered air exposure

Mechanistic pathways were first investigated by analyses of pooled samples on a group level to look for indications of global protein changes using a shotgun proteomic hypothesis-generating method. Findings from the shotgun analysis were further investigated by analyses of individual samples using a targeted label-free analysis with relative quantification of all measured proteins and with absolute quantification of five specific proteins that had been indicated by the shotgun analysis to be associated to the exposure (Fig. 2).

The total protein content in each nasal lavage fluid sample was determined using a BCA protein assay kit.

#### Analysis of pooled samples with discovery shotgun method

Equal amounts of total protein (50 µg) were pooled from all subjects with samples collected at all time points (n = 9), resulting in six pooled samples. The six samples resulted from the samples collected at the three time points (NL 1, NL 2, and NL 3) and for both types of exposure (welding fume exposure or filtered air exposure) (Fig. 1). Samples were reduced with DTT (1.25 mg/mg protein) for 1 h at 60 °C and then alkylated with iodacetamide (2.5 mg/mg protein) for 30 min at room temperature and in darkness. Samples were desalted in centrifugal filters (cut-off 3 kDa) at 4500×*g* for 2 h and then washed with 300 µl 50 mM ammonium acetate three times followed by centrifugation at 4500×*g* for 30 min. Proteins were then trypsin digested (1:20 trypsin:protein) overnight at 37 °C with Promega trypsin and 1 mM calcium chloride.

#### Instrumentation and data analysis

Protein digests (250 ng/injection) were loaded into a nano liquid chromatography system (EASYnLC, Thermo Scientific, Waltham, MA, USA) with a C18 column (100 mm × 0.75 μm, Agilent Technologies, Santa Clara, CA, USA) coupled to a high-resolution mass spectrometer (Orbitrap Velos Pro, Thermo Fisher). Peptides were separated using a 70 min increase from 2 to 40% ACN, supplemented with 0.1% FA, followed by an increase to 90% ACN for 10 min. Peptides were analyzed by a data-dependent acquisition method utilizing collision-induced dissociation for sequencing on an LTQ Velos Orbitrap Pro mass spectrometer (Thermo Scientific). Identification and relative quantification was performed with the MAXQuant software against a human database downloaded from Uniprot (October 31, 2015). Protein levels were determined using label-free quantification based on peptide intensity with at least two peptides (unique and razor, with at least one unique peptide). Relative protein quantification was performed by normalizing each protein level against the protein level found in the baseline sample (the nasal lavage sample collected before exposure) for each exposure, thus the ratios were NL2/NL1 and NL3/NL1, respectively. For a protein to be further evaluated with the targeted method, it had to be detected in at least 50% of the samples (in at least 3 of 6) and have a ratio for being categorized as an increased or decreased protein level of > 1.3 or < 0.8, respectively. When selections according to these criteria were made, a total of 137 welding-associated proteins were identified (Additional file 1). These proteins were further analyzed with IPA (Additional files 2, 3, 4).

#### Analysis of individual samples with the targeted SRM method

The samples were evaporated and dissolved in 50 mM ammonium acetate to a concentration of 400 µg/mL. Proteins were reduced, alkylated, and trypsin digested. All samples were spiked with isotopically labeled standards from matrix metalloproteinase (MMP)9 (4 fmol/µL), alpha-1-antitrypsin (A1AT) (4 fmol/µL), alpha-2-macroglobulin (A2MG) (4 fmol/µL), apolipoprotein (APO)B (4 fmol/µL), and elastin (120 fmol/µL). The digested samples were desalted on a solid-phase extraction column (Evolute ABN 25 mg packing material, 1 mL column, Biotag, Uppsala, Sweden) by loading the digested sample onto the column according to the instructions from the manufacturer. The peptides were eluted with a gradient of 200 mL 30% ACN, 40% ACN, 70% ACN (and 0.1% FA), and 90% ACN, and the solution was evaporated to dryness (SpeedVac, HT-4X Evaporator, Genevac Inc, USA) and reconstituted in 200 μL 0.1% FA (≈ 1 mg/mL). Samples were analyzed twice (20 μg protein per injection). To avoid systematic bias, all samples originating from the same subject but from different time points were prepared and analyzed at the same time.

#### Instrumentation

The SRM analyses were conducted using a 5500 QTRAP hybrid triple quadrupole/linear ion trap mass spectrometer equipped with a TurboIonSpray source (Applied Biosystems/MDS Sciex, Framingham, MA, USA) connected to an LC system (UFLCXR; Shimadzu Corporation, Kyoto, Japan; LC–SRM–MS). The peptides were separated on a C18-column (XBridge peptide BEH C18, 130 Å, 3.5 µm particles, 2.1 mm × 100 mm, Waters Corporation, Milford, Massachusetts, USA) in a 32-min run using a gradient flow of 0.4 mL/min with 0.1% FA in water (A) and 0.1% FA in ACN (B) as mobile phases. The LC gradient was increased from 5 B to 10% B for 10 min, from 10 B to 25% B for 15 min, and from 25 B to 95% B for 1 min where it was kept for 2 min. The column was recalibrated to 5% B for 4 min before the next sample injection.

### SRM assays

#### Selection of SRM assays

Addition of nasal lavage protein SRM assays were added to a method already developed in a previous study [22]. In total, SRM assays for 77 proteins were used from the original SRM method, and new SRM assays were developed for an additional 53 proteins (Additional file 5). Unique peptides were chosen for the SRM assays with at least three transitions per peptide and 1–4 assays per protein were used. The tool UniMaP (http://bioserver-1.bioacademy.gr/Bioserver/UniMaP/Tool_index.php) was used to control if all selected peptides were unique for the targeted protein. The added SRM assays were generated by measuring nasal lavage protein digests in full scan and product ion scan with LC–SRM–MS/MS with a dwell time of 10, 4, and 3 ms. Collision energies were generated in Skyline software according to the earlier developed protocol [22]. Robust transitions with high intensity and good reproducibility [less than 20% coefficient of variation (CV)] were further selected.

The final SRM method contained 256 precursors with 811 transitions targeting 130 proteins and 4 in vivo peptide degradation products from collage 4A1 and elastin (the peptide degradation products were previously determined [23, 24]) (Additional file 6).

#### Selection and optimization of SRM assays for proteins selected for absolute quantification

The proteins MMP9, A1AT, APOB, A2MG, and elastin were selected for absolute quantification. The peptides selected to represent the protein were chosen based on a minimal probability to be missed or partly cleaved or to be biologically or chemically modified [25]. One peptide for each protein was selected, and this peptide (the standard peptide) and the corresponding isotope-labeled peptide (to be used as the internal standard) were purchased from New England Peptide (Gardner, MA, USA).

The collision energy was optimized, and the ion fragments with the highest intensity were selected (Additional file 7). Sensitivity for the selected SRM transitions was assessed by determining the peak area of the quantifier ion of the targeted peptide after injecting 100 fmol of the standard and the internal standards for MMP9, A1AT, A2MG, APOB, and elastin via infusion. The impact of the matrix was assessed by comparing the chromatographic peaks of the transitions in the presence and absence of biological matrix (pooled nasal lavage from 30 volunteers) to assess whether tryptic peptides of nasal lavage proteins interfered with the intended target peptides.

#### Absolute quantification of five proteins

Calibration curves with and without biological matrix were prepared at concentrations of 30, 23, 15, 7.6, 3.8, 1.5, 0.76, 0.38, 0.076, and 0 fmol/µL. The samples for each calibration point were prepared separately and analyzed twice by LC–MS/MS. All calibration samples were spiked with isotopically labeled standards for MMP9 (4 fmol/µL), A1AT (4 fmol/µL), A2MG (4 fmol/µL), APOB (4 fmol/µL), and elastin (120 fmol/µL) (Additional file 8).

#### Determination of the limit of detection (LOD)

LOD was determined by calculating the concentration corresponding to three times the standard deviation of the peak area ratios between the peak area at noise level and the peak area of each corresponding internal standard of eight blank samples and thus LOD in this case corresponds to the noise level that prevailed.

### Reproducibility of the method

The stability of the sample preparation and the reproducibility of the mass spectrometry analyses were determined by analysis of quality control samples. The samples were composed of pooled nasal lavage samples spiked with the different isotopically labeled peptides MMP9 (4 fmol/µL), A1AT (4 fmol/µL), A2MG (4 fmol/µL), APOB (4 fmol/µL), and elastin (120 fmol/µL) (Additional file 9).

### Data processing of individual samples

Data were processed in an automated manner with the Skyline software version 3.5. Peaks were integrated automatically with the algorithm of the Skyline software.

#### Relative quantification

In total, 256 peptides measuring 130 proteins and 4 in vivo peptide degradation products were analyzed on the individual level with targeted SRM. The isotopically labeled standards were used as global normalizers for systemic error correction. The total peak area of each SRM assay was normalized to the total peak areas of each isotopically labeled reference peptide MMP9, A1AT, APOB, and A2MG, respectively (the isotopically labeled peptide for A2MG was represented by both double (927 *m/z*) and triple charged (618 *m/z*) precursors, using each of the precursors as a global normalizer separately). Peptides were thus normalized with all four different isotope-labeled peptides, and data from each normalization step and the non-normalized data were statistically evaluated separately.

#### Absolute quantification

Absolute quantification of MMP9, A1AT, APOB, A2MG, and elastin was determined by calculating the ratio between the total peak areas of the standard peptide and the corresponding labeled peptide (the internal standard). The concentration of the peptide in the sample was calculated by dividing the ratio by the slope of the calibration curve.

### Statistical analysis of individual samples

Protein level was determined in samples obtained from welding fume exposure and from filtered air exposure. To assess the changes at the proteome level, the statistical analyses were conducted on the peptide level and further summarized on the protein level. Data were analyzed with SPSS for Windows 23.0 (SPSS Inc., Chicago, IL, USA). Statistical significance was defined as *p* < 0.05. The data were statistically analyzed with linear mixed modeling (LMM) and Wilcoxon signed rank test.

#### Linear mixed modeling

To assess if the changes in protein levels were statistically significant, the data were analyzed with LMM, a method in which several variables are considered simultaneously, e.g. the time course of the study and the involvement of multiple experimental exposure factors [26].

The dependent variables in LMM were the natural logarithmically transformed total peak area normalized to the isotopically labeled reference peptide of MMP9, A1AT, APOB, A2MG, respectively, for the relative quantification. Fixed effects in the model were the two different exposure types (welding fumes and filtered air) and each time point of sampling (immediately after exposure and the day after exposure). Subject was set to a random effect. The sample taken before exposure was set as a covariate. The repeated covariance types chosen were the unstructured covariance structure, the first-order factor analytic covariance structure, and the compound symmetry covariance structure. The assumption of normally distributed residuals was statistically tested with the Shapiro–Wilk test. The normal distribution of the residuals was used to evaluate the validity of the assumptions of the statistical model and was used as a tool for model selection in combination with Akaike information criterion, which is a measure of the relative quality of a statistical model for a given set of data. Data were analyzed according to following model.

$$Log\,\left( {peptide} \right) = Over\,all\,mean + Exposure + Time$$

Peptide changes were shown by the estimated marginal means of the main effect between the exposure term.

The residual was not normally distributed for all peptides for the relative quantification, and the dataset was therefore further analyzed with an additional non-parametric test to additionally check if the significantly changed proteins could be detected with a less sensitive model.

The concentrations obtained by absolute quantification were natural logarithmically transformed and used as the dependent variable. The data were normally distributed, therefore no more statistical analyses were used for the absolute quantification of the data.

#### Wilcoxon signed rank test

The Wilcoxon signed rank test was conducted for the relative quantification of the different peptides between the different exposure groups at the different time points, calculating the difference between NL1 and NL2 and between NL1 and NL3 for each peptide for each group.

#### False discovery rate (FDR) analysis

Control for multiple testing (8 tests × 276 peptides = 2208 tests for the LMM main effect test, 6 tests × 2 time points × 276 peptides = 3312 tests for the Wilcoxon signed rank test, and FDR-level: 0.05) was adjusted for with sequential goodness of fit for multiple testing using the SGoF+ algorithm [27–29], which resulted in an adjusted significance level 0.030 for the LMM test and 0.036 for the Wilcoxon signed rank test. The multiple test adjustment was conducted with the software SGoF+ (20) (downloaded from http://webs.uvigo.es/acraaj/SGoF.htm) and with the user-defined significance level set at *p* < 0.05.

#### Spearman rank correlation

Associations between MMP9 concentration and FEV_1_% and FVC% were examined using Spearman’s rank correlation. Statistical significance was defined as *p* < 0.05 (two-sided *p* values were determined).

### IPA analysis

To evaluate common interactions and pathways of the identified proteins with changed levels, the results were further analyzed with IPA software (Ingenuity Systems, Redwood City, CA, USA, www.ingenuity.com). Significantly changed proteins were mapped and compared to known pathways, diseases, functions, and connecting regulators. The data were also evaluated based on upstream regulators with positive z-scores (activating capacity) and negative z-scores (inhibiting capacity). Key regulators are proteins with an earlier established association with the activation or inhibition of many of the identified proteins, but the regulators are not necessarily detected themselves in the analysis. Default settings were used except for species, which was set to human. In addition, only experimentally observed relationships were considered.

## Results

### Shotgun data analysis of pooled samples

#### Protein identification and relative quantification

The six pooled samples from the welders were analyzed by shotgun LC–MS/MS. In total, 336 proteins were identified in the pooled samples, whereof 137 proteins remained when the criteria described in the experimental section were applied (Additional file 1). The first observations (NL2/NL1) and second observations (NL3/NL1) were further analyzed with IPA for selection of candidate proteins to be included with targeted SRM (Additional files 2, 3, 4).

#### Pathway analysis of proteins identified with shotgun analysis and selection of proteins to be further analyzed with SRM

The pathway analysis indicated several pathways associated with the exposure. These were activation of the LXR/RXR pathway in monocytes, the inflammatory defense mechanism in macrophages, the coagulation system, and anti-inflammatory mechanisms, all of which influence the status of the extracellular matrix (ECM) (Additional files 2, 3). From these pathways, five representative proteins were selected for comprehensive quantitative data collection, including MMP9, which was identified as a key connecting mediator (Additional file 3); A1AT and A2MG, two anti-inflammatory proteins associated with MMP9 activity; APOB, an important lipid-loading protein involved in the low density lipoprotein protein complex; and elastin, an important extracellular protein involved in sustaining the elasticity of the ECM.

Proteins with currently unknown biological functions (according to IPA) were excluded from further analysis. In total, 97 proteins remained in the candidate list to be further analyzed with the targeted SRM method on the individual level. To enable a comprehensive monitoring of biological processes, proteins theoretically involved in the above-mentioned pathways were added to the candidate list. Finally, 150 protein candidates and 4 in vivo degradation peptides from collagen 4A1 and elastin remained in the list (Additional file 4). SRM assays were developed for these candidates (Additional files 5, 6).

In order to further investigate the findings from the analysis of pooled samples, individual samples were analyzed with SRM.

## SRM analysis of individual samples

### Statistical evaluation

#### Relative quantification of individual samples using LMM

Forty-six significantly changed proteins were found to be associated with welding fume particle exposure compared to the filtered air (Table 1). The beta value reflects the average effect change constant across the individuals between the exposures to welding fumes and filtered air. After the multiple statistical tests were controlled for FDR, 32 proteins were still significantly changed.

**Table 1:** Significantly changed proteins from the individual samples evaluated by LMM

AC	Gene name	Protein names	Beta value	CI 95% (min–max)	FDR	Sig. peptides	Nr. peptides
*Increased*							
P04040	CATA	Catalase	2.42	(1.46–4.02)		1	2
P02766	TTHY	Transthyretin	1.38	(1.02–1.86)	–^f^	1	2
P15924	DESP	Desmoplakin	1.37	(1.05–1.77)		1	2
A6NC86	PINLY	Phospholipase A2 inhibitor	1.35	(1.09–1.66)		1	1
P80188	NGAL	Neutrophil gelatinase-associated lipocalin	1.35	(1.02–1.78)	–^f^	2	3
P18510	IL1RN	Interleukin-1 receptor antagonist protein	1.30	(1.02–1.67)	–^f^	1	1
Q8TDL5	LPLC1	BPI fold-containing family B member 1	1.29	(1.03–1.61)	–^f,f^	2	2
P0DJI8	SAA1	Serum amyloid A-1 protein	1.28	(1.00–1.62)	–^f^	1	2
P01023	A2MG	Alpha-2-macroglobulin	1.26	(1.10–1.45)		1	4
P02790	HEMO	Hemopexin	1.26	(1.05–1.51)		1	3
Q08380	LG3BP	Galectin-3-binding protein	1.26	(1.10–1.44)		1	2
P01834	IGKC	Immunoglobulin kappa constant	1.24	(1.04–1.48)		2	2
P05155	IC1	Plasma protease C1 inhibitor	1.23	(1.02–1.50)	–^f^	1	3
P08603	CFAH	Complement factor H	1.23	(1.03–1.48)		1	1
P02760	AMBP	Protein AMBP	1.23	(1.04–1.46)		1	2
Q92743	HTRA1	Serine protease HTRA1	1.23	(1.10–1.37)		1	2
P22894	MMP8	Neutrophil collagenase	1.22	(1.08–1.38)		1	1
P07737	PROF1	Profilin-1	1.20	(1.00–1.43)	–^f^	1	1
P27169	PON1	Serum paraoxonase	1.20	(1.11–1.29)		1	3
P10909	CLUS	Clusterin	1.19	(1.11–1.28)		2	3
Q96DA0	ZG16B	Zymogen granule protein 16 homolog B	1.18	(1.03–1.37)		1	1
P04792	HSPB1	Heat shock protein beta-1	1.16	(1.01–1.34)	–^f^	1	2
*Decreased*							
P07602	SAP	Prosaposin	0.92	(0.88–0.97)		1	3
Q9GZX6	IL22	Interleukin-22	0.88	(0.79–0.99)	–^f^	1	3
P01034	CYTC	Cystatin-C	0.88	(0.79–0.97)		1	1
Q9Y624	JAM1	Junctional adhesion molecule A	0.87	(0.77–0.97)		1	2
Q13822	ENPP2	Ectonucleotide pyrophosphatase	0.85	(0.74–0.97)		1	1
O75223	GGCT	Gamma-glutamylcyclotransferase	0.82	(0.73–0.93)		1	3
P37802	TAGL2	Transgelin-2	0.82	(0.70–0.95)		1	1
Q14118	DAG1	Dystroglycan	0.81	(0.66–1.00)	–^f^	1	2
P04080	CYTB	Cystatin-B	0.81	(0.71–0.92)		1	1
P02751	FINC	Fibronectin	0.80	(0.66–0.97)		1	3
P14780	MMP9	Matrix metalloproteinase-9	0.78	(0.62–0.97)	–^f^	2	3
P31944	CASPE	Caspase-14	0.77	(0.62–0.95)		2	2
P05164	PERM	Myeloperoxidase	0.75	(0.62–0.92)	–^f^	2	2
Q14019	COTL1	Coactosin-like protein	0.72	(0.58–0.89)		1	2
P06727	APOA4	Apolipoprotein A-IV	0.71	(0.54–0.93)		1	3
P01033	TIMP1	Metalloproteinase inhibitor 1	0.71	(0.57–0.88)		1	2
O75594	PGRP1	Peptidoglycan recognition protein 1	0.70	(0.54–0.90)		3	3
P29401	TKT	Transketolase	0.69	(0.50–0.94)		1	2
P04406	G3P	Glyceraldehyde-3-phosphate dehydrogenase	0.68	(0.57–0.81)		1	1
O75556	SG2A1	Mammaglobin-B	0.67	(0.47–0.96)	–^f^	1	3
P02749	APOH	Beta-2-glycoprotein 1	0.65	(0.49–0.86)	–^f^	2	2
P01008	ANT3	Antithrombin-III	0.53	(0.31–0.89)		1	1
Q6UWW0	LCN15	Lipocalin-15	0.43	(0.25–0.72)		1	2
P08670	VIME	Vimentin	0.72	(0.54–0.95)		1	2

Relative quantification of individual samples generated this protein list of the significantly changed proteins after welding fume exposure compared to filtered air, and these were evaluated by LMM, *p* < 0.05. The beta value represents the average change of the protein level after welding fume exposure

^f ^Peptides not significantly changed after multiple test correction (FDR), *p* > 0.03. *Sig. peptides* number of significantly changed peptides. *Nr. Peptides* number of peptides used in the SRM method representing the specific protein

#### Relative quantification and statistical analysis with the Wilcoxon signed rank test

In total, 56 proteins were significantly changed when using the Wilcoxon signed rank test to compare the changes of peptide levels between the groups at the different time points (Table 2). After adjustment for FDR, 35 proteins still remained significantly changed.

**Table 2:** Significantly changed proteins from the individual samples evaluated with Wilcoxon signed rank test

AC	Gene name	Protein name	After exposure	Day after exposure	Significant peptides	FDR
*Proteins increased immediately after exposure*						
P02765	FETUA	Alpha-2-HS-glycoprotein	Increase		1	–^f^
*Proteins increased immediately after exposure and the day after exposure*						
A6NC86	PINLY	Phospholipase A2 inhibitor	Increase	Increase	1	
P01019	ANGT	Angiotensinogen	Increase	Increase	1	–f
P01834	IGKC	Immunoglobulin kappa constant	Increase	Increase	2	
P01857	IGHG1	Immunoglobulin heavy constant gamma 1	Increase	Increase	1	
P02768	ALBU	Serum albumin	Increase	Increase	2	
P26038	MOES	Moesin	Increase	Increase	1	
Q92743	HTRA1	Serine protease HTRA1	Increase	Increase	1	
*Proteins increased the day after exposure*						
P01023	A2MG	Alpha-2-macroglobulin		Increase	2	
P01859	IGHG2	Immunoglobulin heavy constant gamma 2		Increase	1	–^f^
P02760	AMBP	Protein AMBP		Increase	1	–^f^
P02787	TRFE	Serotransferrin		Increase	1	–^f^
P02790	HEMO	Hemopexin		Increase	1	
P04040	CATA	Catalase		Increase	1	
P04792	HSPB1	Heat shock protein beta-1		Increase	1	
P07737	PROF1	Profilin-1		Increase	1	–^f^
P08123	CO1A2	Collagen alpha-2		Increase	1	–^f^
P08603	CFAH	Complement factor H		Increase	1	
P15924	DESP	Desmoplakin		Increase	1	–^f^
P18510	IL1RA	Interleukin-1 receptor antagonist protein		Increase	1	–^f^
P27169	PON1	Serum paraoxonase		Increase	1	
P31947	1433S	14-3-3 protein sigma		Increase	1	
P36955	PEDF	Pigment epithelium-derived factor		Increase	1	
Q04118	PRB3	Basic salivary proline-rich protein 3		Increase	1	–^f^
Q07157	ZO1	Tight junction protein ZO-1		Increase	1	–^f^
P11684	UTER	Uteroglobin		Increase	2	
*Proteins decreased immediately after exposure but increased the day after exposure*						
P07602	SAP	Prosaposin	Decrease	Increase	2	–^f,f^
Q96IY4	CBPB2	Carboxypeptidase B2	Decrease	Increase	2	
P01877	IGHA2	Immunoglobulin heavy constant alpha 2	Decrease	Increase	1	–^f^
*Proteins decreased immediately after exposure *						
O75556	SG2A1	Mammaglobin-B	Decrease		1	
P01008	ANT3	Antithrombin-III	Decrease		1	
P01034	CYTC	Cystatin-C	Decrease		2	
P01036	CYTS	Cystatin-S	Decrease		1	
P04406	G3P	Glyceraldehyde-3-phosphate dehydrogenase	Decrease		1	
P0DMV9	HS71B	Heat shock 70 kDa protein 1B	Decrease		1	
P13796	PLSL	Plastin-2	Decrease		1	
P14780	MMP9	Matrix metalloproteinase-9	Decrease		1	
P15311	EZRI	Ezrin	Decrease		1	
P15502	ELN	Elastin	Decrease		1	
P37802	TAGL2	Transgelin-2	Decrease		1	
Q13822	ENPP2	Ectonucleotide pyrophosphatase	Decrease		1	
Q14019	COTL1	Coactosin-like protein	Decrease		1	
Q14508	WFDC2	WAP four-disulfide core domain protein 2	Decrease		1	
Q9GZZ8	LACRT	Extracellular glycoprotein lacritin	Decrease		1	
Q9Y624	JAM1	Junctional adhesion molecule A	Decrease		1	
P04080	CYTB	Cystatin-B	Decrease		1	
Q6UXB2	VCC1	C-X-C motif chemokine 17	Decrease		1	
Q6P5S2	CF058	Protein LEG1 homolog	Decrease		2	–^f,f^
*Proteins decreased immediately after exposure and the day after exposure*						
O75594	PGRP1	Peptidoglycan recognition protein 1	Decrease	Decrease	2	
P05164	PERM	Myeloperoxidase	Decrease	Decrease	2	
P06727	APOA4	Apolipoprotein A-IV	Decrease	Decrease	1	–^f^
*Proteins decreased the day after exposure*						
P02749	APOH	Beta-2-glycoprotein 1		Decrease	1	–^f^
P02751	FINC	Fibronectin		Decrease	1	
P08311	CATG	Cathepsin G		Decrease	1	
P20061	TCO1	Transcobalamin-1		Decrease	1	–^f^
Q6UWW0	LCN15	Lipocalin-15		Decrease	1	–^f^

Relative quantification of individual samples generated this list of the significantly changed proteins after welding fume exposure compared to filtered air, and these were statistically evaluated with the Wilcoxon signed rank test both immediately after exposure and the day after exposure, *p* < 0.05

^f ^Peptides not significantly changed after multiple test correction (FDR), *p* > 0.036

#### Absolute quantification of individual samples using LMM

Quantitative data could only be obtained for MMP9, A1AT, and A2MG (Additional file 9). LODs were determined for the proteins in samples without nasal lavage matrix (Table 3).

**Table 3:** Absolute quantification of individual sample

Protein	Mean slope in NL	Mean slope in MilliQ water	LOD (fmol/µL)	Exposure	Mean; median; (max–min) concentration (fmol/µL)			Statistical evaluation		
					Before exposure	After exposure	Day after exposure	Beta-value	95% CI	*p* value
MMP9	0.291	0.275	0.043	A	0.26; 0.14; (0.02–0.61)	0.14; 0.13; (0.02–0.37)	0.19; 0.13; (0.02–0.36)	0.77	0.63–0.93	0.015
				B	0.16; 0.15; (0.02–0.42)	0.16; 0.11 (0.03–0.52)	0.18; 0.16; (0.08–0.4)			
A1AT	0.153	0.158	0.205	A	0.51; 0.37; (0.23–1.28)	0.50; 0.37; (0.23–1.6)	0.44; 0.52; (0.14–0.94)	1.20	0.77–1.87	0.369
				B	0.67; 0.51; (0.3–2.12)	0.52; 0.41; (0.21–1.84)	0.56; 0.45; (0.2–1.48)			
A2 MG	0.184	0.153	0.075	A	0.52; 0.43; (0.09–0.13)	0.42; 0.20; (0.08–1.32)	0.35; 0.29; (0.05–0.95)	0.95	0.73–1.23	0.669
				B	0.49; 0.32; (0.16–1.13)	0.47; 0.24; (0.09–1.55)	0.48; 0.51; (0.11–1.44)			
APOB	0.164	0.177	0.356	A/B	NA	NA	NA	–	–	–
Elastin	0.432*	0.339	0.339	A/B	NA	NA	NA	–	–	–

Mean slope of the calibration curve for each quantitative protein and the calculated LOD (determined from blank samples)

*NL* nasal lavage, *A* welding fume particle exposure, *B* filtered air exposure, *NA* no quantitative data could be obtained

* Big variation between the slopes

The absolute quantification of the individual samples showed that 90% of all samples had a concentration higher than the LOD for A1AT, 95% of all samples had a concentration higher than the LOD for A2MG, and 88% of all samples had a concentration higher than the LOD for MMP9 (Table 3). The anti-inflammatory proteins A1AT and A2MG were found in higher amounts than MMP9 in all samples (Table 3). The only protein that was significantly changed due to welding fume exposure compared to the filtered air was MMP9. MMP9 had a beta value of 0.77, indicating a decrease in the protein level (Table 3).

#### MMP9 levels and lung function

**Fig. 3:**
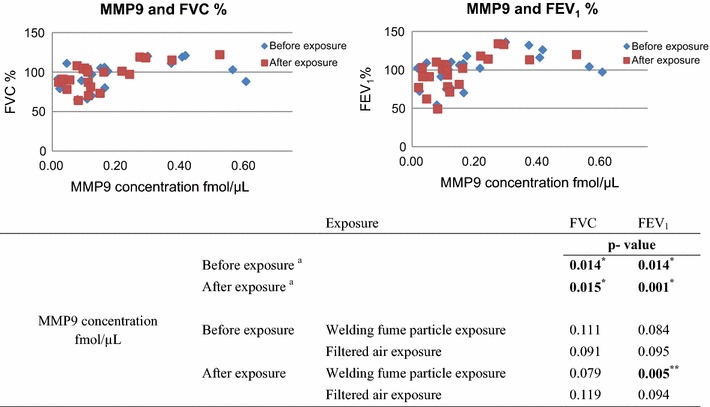
Correlation between the MMP9 concentration (fmol/µL) and lung function (FVC and FEV_1_%) ^a^Combining the data from both the welding fume exposure day and filtered air exposure day. *Correlation is significant at the 0.05 level (2-tailed). **Correlation is significant at the 0.01 level (2-tailed)

The relationships between MMP9 concentration and lung function were evaluated. Combining the data of the welding fume particle and filtered air exposure for MMP9 concentration and the lung function, revealed that the levels of MMP9 were significantly associated with FVC (*p* = 0.014) and FEV_1_ (*p* = 0.014) before exposure and with FVC (*p* = 0.015) and FEV_1_ (*p* = 0.001) after exposure (Fig. 3). MMP9 had no significant association with FVC or FEV_1_ when separating the welding fume particle and filtered air exposure data in the statistical test for the data assessed before exposure. The levels of MMP9 in nasal lavage samples collected after exposure to welding fume particles were significantly associated with FEV_1_ (*p* = 0.005), and a trend toward an association with FVC (*p* = 0.079) was seen. The welders with the lowest FVC and FEV_1_ had low levels of MMP9 compared to the rest of the welders in this study (Additional file 10). The welders with the highest FVC and FEV_1_ during the baseline medical examination had high levels of MMP9 compared to the rest of the welders in this study.

### Quality of the SRM data

Duplicate analyses of 126 prepared samples were performed by LC-SRM. Based on analyses of all samples, 69% of the SRM assays had a CV less than 20%. When normalizing the total area of each SRM assay to the total area of either of the isotopically labeled peptides belonging to MMP9, A1AT, A2MG, and APOB, 84, 83, 72, 73, and 84%, respectively, had a CV less than 20%.

### Pathway analysis of significantly changed proteins from individual samples

Pathway analysis of the proteins with significantly changed levels according to LMM suggested that the proteins had two major pathways in common (Table 4). The first pathway was activation of acute phase response signaling, and this was supported by protein data indicative of induced inflammation, including acute phase proteins such as hemopexin and serum amyloid A1, both of which were upregulated following exposure to welding fumes. The second pathway was activation of LXR/RXR, a nuclear receptor family involved in lipid, inflammation, and cholesterol regulation, and this was supported by decreased levels of MMP9 and by increased levels of paraxonase 1 and clusterin, both of which are proteins found in high-density lipoproteins.

**Table 4:** Pathways associated with welding fume exposure

Symbol	Protein	AC	Expr. fold change	Expected
*LXR/RXR activation (positive z-score 1.26)*				
AMBP	Alpha-1-microglobulin/bikunin precursor	P02760	1.23	Up
APOA4	Apolipoprotein A4	P06727	− 1.41	Up
APOH	Apolipoprotein H	P02749	− 1.54	Up
CLU	Clusterin	P10909	1.19	Up
HPX	Hemopexin	P02790	1.26	Up
IL1RN	Interleukin 1 receptor antagonist	P18510	1.30	Down
MMP9	Matrix metallopeptidase 9	P14780	− 1.29	Down
PON1	Paraoxonase 1	P27169	1.20	Up
SAA1	Serum amyloid A1	P0DJI8	1.28	Up
TTR	Transthyretin	P02766	1.38	Up
*Acute phase response signaling (positive z-score 0.378)*				
A2M	Alpha-2-macroglobulin	P01023	1.27	Up
AMBP	Alpha-1-microglobulin/bikunin precursor	P02760	1.23	Down
APOH	Apolipoprotein H	P02749	− 1.54	Down
FN1	Fibronectin 1	P02751	− 1.25	Up
HPX	Hemopexin	P02790	1.26	Up
IL1RN	Interleukin 1 receptor antagonist	P18510	1.30	Up
SAA1	Serum amyloid A1	P0DJI8	1.28	
SERPING1	Serpin family G member 1	P05155	1.24	
TTR	Transthyretin	P02766	1.38	Down

The significantly changed proteins found with the LMM statistical method showed that these canonical pathways were activated due to welding fume exposure. AC: accession number. Expected: The changed direction of the protein for this canonical pathway to be activated are expected to go in e certain direction when its activated

**Fig. 4:**
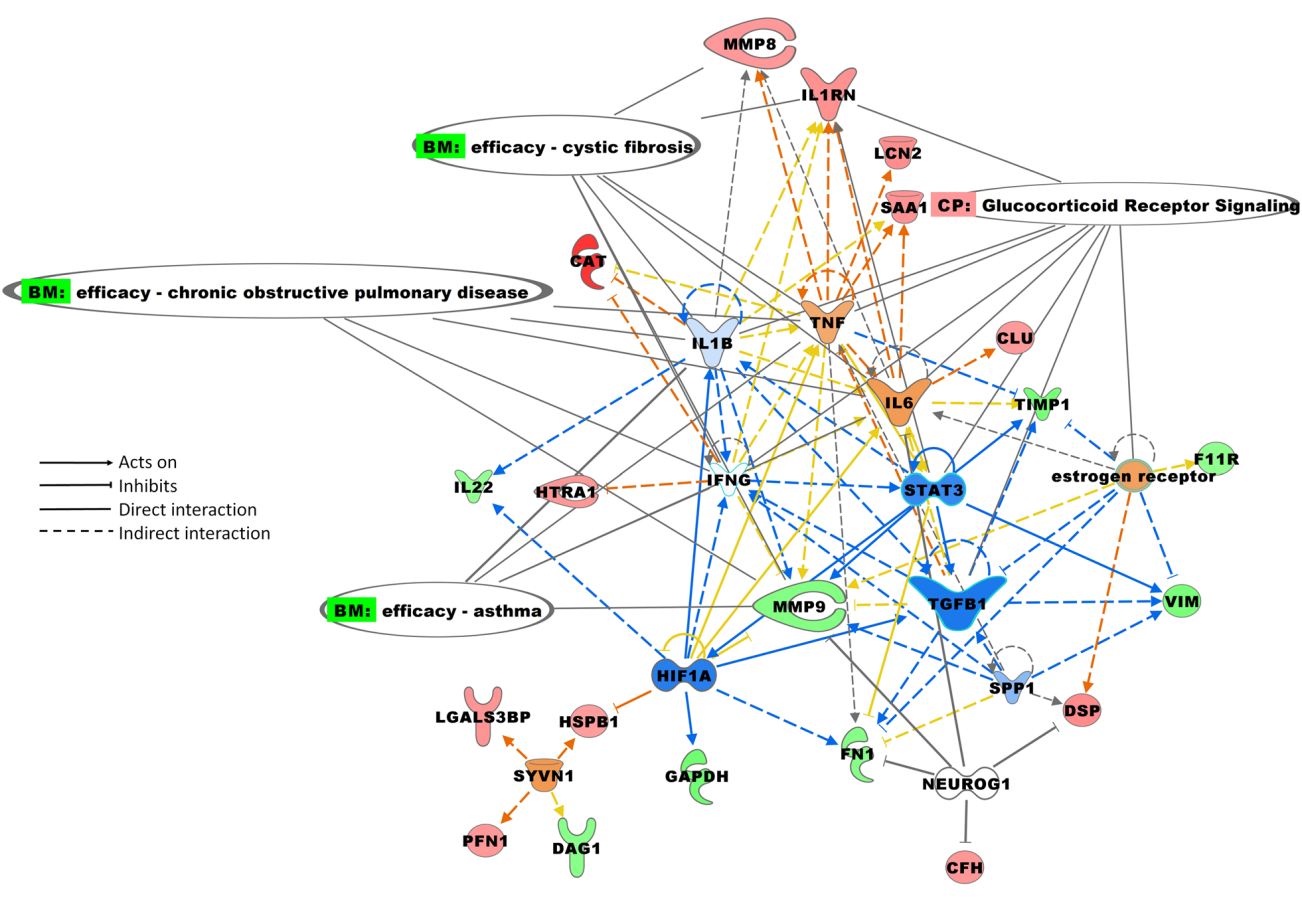
Network of the significantly changed proteins and the upstream regulators. The significantly changed proteins from the LMM table were associated with the upstream regulators IL6, estrogen receptor, H1FA, IFNG, IL1B, NEUROG1, SPP1, SYVN1, TGFB1, TNF, and STAT3. The canonical pathways connected to these molecular regulators included glucocorticoid receptor signaling. Biomarker enrichment of this network connection showed connections to cystic fibrosis, chronic obstructive pulmonary disease, and asthma. Red = increased measurement, Green = decreased measurement, Orange = indicated activated, Blue = indicated inhibited

Based on the significantly changed proteins, a number of key regulators were identified in the IPA analysis (Fig. 4). A key connecting regulator identified in this study was transforming growth factor (TGF)-β, and it was indicated to be inhibited. The following mediators that were detected in our data set supported this finding: MMP9, tissue inhibitor of metalloproteinases 1, fibronectin, and vimentin. Another identified key regulator was IL-6, a pro-inflammatory cytokine, and this was supported by MMP8, serum amyloid A1, interleukin-1 receptor antagonist, and tissue inhibitor of metalloproteinases 1. IL-6 was also associated with TGF-β. TNF and IL-1β were also identified as key regulators in the induced proteome effect. The key regulators and proteins identified in this study have previously been associated with several diseases, including chronic obstructive pulmonary disease, cystic fibrosis, and asthma (Fig. 4).

The significantly changed proteins identified with the Wilcoxon signed rank test also showed activation of the LXR/RXR pathway and activation of acute phase signaling (Additional file 11).

## Discussion

### Biological findings

In this study, we observed the effects on the proteome level in the nasal region after exposure to welding fumes. Proteins with changed levels were detected following welding fume particle exposure compared to filtered air, although neither clinical symptoms nor specific pro-inflammatory markers in nasal lavage were recorded in the study subjects [18]. This suggests that by applying a more comprehensive proteomic analysis, important biological information might still be gained even if no clinical symptoms can be recorded. Overall, the data analysis suggests that the proteins that differed in expression between welding fume particle and filtered air exposure are primarily involved in acute phase response and lipid regulation.

Changes in the proteome might result from a large number of factors, including metabolism and current health status, and the levels of many proteins with regulatory functions show a diurnal variation [30, 31]. By applying a study design where each study subject is its own control and where protein changes are associated with welding fume particle exposure compared to filtered air exposure, any changed levels of proteins will most likely be linked to the external exposure. Diurnal variation of protein levels was thus automatically taken into account. It is possible that previous exposure might influence the symptoms and sub-clinical responses, but in this project exposures were performed on Mondays making sure that no occupational exposure to welding occurred at least 48 h prior to the experimental exposure, minimizing the short term response interference.

In epidemiological studies of welders, the study subjects are exposed to both the emitted particles and the gases making it impossible to determine the exact cause of any biological response. In this study, the gases and particles were separated, and the study subjects were mostly exposed to the particle fraction. Previous studies have shown that when welding fumes are inhaled by humans, an inflammatory response is induced [15]. The result from the present study shows that the welding fume particles alone might induce an inflammatory response on the proteome level.

The subjects were exposed to particles at levels corresponding to a fifth of the permissible occupational exposure limit level in Sweden. The inhaled dose was, however, probably even less during the experimental exposure than it would have been in a true exposure situation because the welders were sitting still in a chamber without any physically demanding tasks. Even with this limitation, the welding fume particle exposure could still induce a protein response in the nasal region of the symptomatic welders.

The pro-inflammatory cytokines TNF, IL-1β, and IL-6 are suggested in this study to be key regulators connecting the proteins with changed levels, and this supports previous findings regarding inflammation as an important mechanism behind the response induced by welding fume exposure [5, 32]. Our data also indicate that the welding fume particle exposure not only affects the inflammatory response, but also induces imbalances in the regulation of the ECM. There a number of structural proteins that form the basis of connective tissue in the respiratory tract, including collagen I A2, desmoplakin, fibronectin, moesin, tight junction protein ZO-1, and junctional adhesion molecule A. The formation and degradation of connective tissue is highly regulated by the enzymatic activity of proteases. Significantly changed levels of several structural proteins were found following welding fume particle exposure, many with increased levels the day after exposure. Also, significantly changed levels of proteases such as metalloproteinases and a serine protease HTRA1, were detected. MMP8 increased in nasal lavage after exposure, and MMP9 decreased after exposure. MMP8 and MMP9 belong to a family of proteases that degrade ECM proteins. They play a central role in normal tissue structure to maintain the balance between the formation and degradation of ECM proteins and are involved in respiratory tract remodeling [33, 34]. MMPs are in turn inhibited by tissue inhibitor of metalloproteinases [35]. TGF-β was identified in the IPA analysis (Fig. 4), and this cytokine activates fibroblasts to produce collagen [36] and thus plays an important role in tissue repair by regulating the activities of proteins involved in the structural formation of connecting tissue as well as inflammatory responses.

The increased or decreased levels of structural proteins in nasal lavage fluid might be the result of tissue damage induced by the exposure, such that damaged cells release their intracellular content; inflammatory or epithelial cells secreting extracellular components and cleaving the structural proteins; or damage to the tight junctions of the epithelial cells such that the permeability increases.

Previous studies show an association between welding fume exposure and increased risk of developing fibrosis, chronic obstructive pulmonary disease, and asthma [37–39], and MMPs have been suggested to play an important role in the pathogenesis of these respiratory diseases (Fig. 4). MMPs can show both pro- and anti-fibrotic activity by degrading ECM proteins and activating various signaling molecules that influence the inflammatory response as well as through inhibition of ECM degradation processes [40–42]. Chronic obstructive pulmonary disease and asthma are both associated with airflow restriction and progressive remodeling, which affect the respiratory tract, although the localization and character of the structural changes throughout the respiratory tract wall are different [43]. The remodeling concerns highly composed structural transformation, which affects the airways and leads to significant functional impairment [44].

In normal tissue, the balance between the formation and degradation of ECM proteins is strictly controlled to maintain tissue structure and function [45]. If this balance is disrupted, accumulation of structural connective tissue can lead to fibrotic tissues. Defined by the accumulation of ECM proteins, fibrosis results in scarring and thickening of the affected tissue; it is in essence an exaggerated wound healing response that interferes with normal organ function. In this study, a significant correlation between the concentration of MMP9 in nasal lavage and lung function (FEV_1_ and FVC%) was detected (Fig. 3). A relationship between MMP9 concentration and pulmonary function, bronchodilator response, and emphysema severity has previously been suggested [46, 47]. Together, these results suggest that the MMP9 level might be associated with the lung function status of each individual welder in this study. In a previous study of welders with and without symptoms, gene expression analysis in induced sputum [48] revealed that genes differentially expressed following welding fume exposure were connected to responses related to tissue damage, immune defense, and inflammation, and one gene that was highly expressed by the asthmatic subjects codes for MMP25, a membrane bound metalloproteinase. Prolonged or frequent exposure to pro-inflammatory agents might promote the development of chronic inflammation with associated damage and transformation of the respiratory epithelium [49, 50].

Also interesting is the presence and increase of the high density lipoprotein-associated proteins serum amyloid A1, paraxonase 1, and clusterin. Knowledge is limited regarding the presence of high density lipoprotein in nasal mucosa, but in the blood stream serum amyloid A1 is an important acute phase protein, and paraxonase 1 is an important antioxidant [51, 52]. Increased paraxonase 1 levels after welding fume exposure might reflect increased oxidative stress in the mucosa and thereby an increased need for antioxidant activities. Regarding clusterin, it has previously been shown that the human clusterin gene is responsive to acute in vivo oxidative stress induced by heavy metals [53]. However, these suggestions have to be further studied because there is a possibility of leakage from the circulation as result of exposure-related tissue damage. A previous study [18] of a symptomatic welding group showed that they had an increasing trend of blood neutrophils after exposure and that they had a tenfold higher level of exhaled breath condensate leukotriene B4 compared to the other groups. Leukotrienes use lipid signaling to convey information order to regulate immune responses, and leukotriene B4 is involved in inflammation [54]. Its primary function is to recruit neutrophils to areas of tissue damage, though it also helps to promote the production of inflammatory cytokines by various immune cells.

Previous studies have shown that most of the upregulated inflammatory response was diminished within 19 h after exposure to welding fumes [55]. This study showed that the inflammatory response that results from welding fume particle exposure is still detectable the day after exposure and that the levels of proteins in the ECM were affected. In parallel, immunosuppressive mediators (A2MG, interleukin-1 receptor antagonist, and uteroglobin) were also detected the day after exposure suggesting that processes attempting to resolve the inflammation had been initiated. If the inflammation is still present the day after exposure and the exposure continues, this might lead to a prolonged inflammatory response with no time to resolve itself.

### Methodological considerations

#### Discovery followed by a targeted mass spectrometry method

More than 300 proteins were identified with the shotgun analysis. Cutoff criteria were used to minimize labor and the time-consuming analysis of redundant proteins. To focus on the proteins that are changed on global level, the median ratio of NL2/NL1 and NL3/NL1 for the increased or decreased proteins was chosen as the cutoff for proteins considered to have a relatively large enough change to detect. The chosen cutoffs were based on the magnitude of change detected for this set of data, which could differ depending on the exposure and the studied group. The combination of the discovery shotgun method and targeted mass spectrometry method (Fig. 2) enabled us to focus the measurements on relevant proteins with potential interest in the induced effects. Another advantage was that crucial proteins that for some reason were not detected with the shotgun method were identified via the pathway analysis in the targeted method and were selected for further analysis.

#### Absolute quantification in comparison to relative quantification for individual samples

Statistical analysis of protein levels determined with absolute quantification showed that the level of MMP9 was significantly changed after welding exposure, whereas the statistical analysis of protein levels determined with relative quantification showed that MMP9 as well as A2MG were significantly changed. However, absolute quantification was based on only one peptide, which made this method less robust, although the analysis had high reproducibility. These results show that it is important to include several peptides in each assay for quantification. In shotgun MS identification of a protein is usually based on many peptides. For relative quantitative SRM methods as well, many SRM assays with peptide criteria are included in the measurement of a protein giving a high degree of protein coverage. But since mass spectrometry is not inherently quantitative because of differences in the ionization efficiency and/or detectability of the many peptides in a given sample it cannot determine the absolute abundance of proteins in a sample. The intensity of a peak in a mass spectrum is not a good indicator of the amount of the analyte in the sample, although differences in peak intensity of the same analyte between multiple samples accurately reflect relative differences in its abundance. In this study the absolute quantification was based on peptides as opposed to proteins consequently subjected to trypsin digestion and therefore post-digest addition of internal standards was applied. One limitation with such a strategy is that any loss occurring at the digestion step is not compensated for. It has earlier been shown that losses of peptides may occur during trypsin digestion [56]. For absolute quantification, the selected peptide is also chosen based on several stringent criteria, thus leaving very few peptides selectable for absolute quantification but there is no guarantee that this peptide ionizes well. This might leave us with a peptide with low reproducibility and low sensitivity which will have a major effect on the absolute quantification method. Therefore, the label free relative quantification is advantageous in terms of providing reasonable proteome coverage with only little investment and method optimization, but may be limited in terms of accuracy and selectivity.

#### Verification of protein changes

Different LC–MS techniques were applied in this study to qualitatively and quantitatively determine proteins. A discovery based shotgun LC–MS analysis was used initially to identify proteins on the group level. Then, semi-quantitative and quantitative methods were applied on individual samples to determine and quantify protein levels. The mass spectrometry methodology and especially in combination with LC-separation offers identification and quantification of very high specificity and accuracy. In the SRM assays we included unique peptides, at least three transitions per peptide and at least three assays per protein were used. In addition, an isotope labelled internal standard was included during absolute quantification to further support the specificity. If robust mass spectrometric assays for quantification are available, e.g. as for MMP9 in this study, such methodology may be sufficient to verify proteome changes determined with semi-quantitative mass spectrometry. Should robust quantitative MS assays not be available then intact protein testing using immunoassays may be more suitable to confirm results. However, immunoassay of proteins may suffer from cross-reactivity generating overestimated results and it could be argued that SRM LC–MS is superior to western blot when it comes to accuracy and specificity [57].

#### Global normalization

The strength of this approach was the use of the isotopically labeled peptides as global normalizers for the peptides that were relatively quantified. This was applied to control for variations in sample preparation, desalting, sample handling, and instrument operation. This type of normalization is rather rough, and there is a risk of losing important findings for peptides that do not vary in the same way as the global isotopically labeled peptide. Using more than one isotopically labeled peptide as a global normalizer increases the chance of finding a good normalization for each peptide.

#### Statistical evaluation on the peptide level for individual samples

For most of the significantly changed proteins, only one peptide showed a significantly changed level (Tables 1, 3). The reason for this could be that different signal intensities could be generated from different peptides depending on the ionization capacity of that peptide. A weak signal might cause greater variation and therefore a larger degree of uncertainty. Proteins were represented by 1–4 peptides, and when the trends in the changes in peptide levels representing the same protein were in line with each other, which was the case for a majority of the analyzed peptides, the results can be considered reliable.

Thirty proteins could be identified both with LMM and Wilcoxon signed rank test, and 26 proteins were exclusively identified with the Wilcoxon signed rank (Table 3) and 16 proteins were exclusively identified with LMM. To a large extent, the two statistical models identified similar proteins that were significantly changed upon exposure to welding fumes. The LMM method yielded a higher sensitivity (lower *p* values) for proteins that were significantly changed. LMM can handle several variables in the same statistical analysis, resulting in the need for fewer statistical tests, and thus lower FDR.

#### Pathway analysis

Pathway analysis identifies common signaling molecules shared between the proteins and if several proteins can be identified in the same pathway, then there is a higher likelihood that this pathway is involved in the biological response. The changed direction of the proteins for the identified canonical pathways to be activated are expected to go in e certain direction when its activated. Our data does not fully go in line with this canonical pathway protein direction. The changed protein levels after an exposure might be driven by a cascade of changed proteins [58, 59], with different protein changes appearing at different time points. In this study, the sampling is taken just a few times after the exposure. This will make the appearance of all the altered proteins impossible to find from the few sampling points.

#### Sampling

By instilling the nasal cavity with saline solution, cells and secreted mediators as well as intracellular components released because of cell damage in the nose can be collected. If cells were damaged by the sampling procedure and not by the exposure, this would occur at all sampling times and thus no significant change would be observed between the two exposures. However, if an exposure induces some cell damage so that, for example, the permeability of the nasal mucosa were to increase, this could be observed by detecting components from the mucosal tissue.

## Conclusion

In conclusion, by applying a comprehensive mass spectrometric work flow a proteome-level response in the nasal upper airway region was demonstrated following mild steel welding fume particle exposure even though no clinical symptoms could be detected. Proteins with significantly changed levels were associated with inflammation and lipid signaling pathways and with changes in the status of the ECM. Several proteins, including MMP8 and MMP9, that were associated with the welding fume particle exposure should be further investigated as biomarkers in future studies.

## Additional files

**Additional file 1.** Protein identification and mechanism hypothesis generation from pooled samples. The protein list generated from the analysis of pooled samples. Calculated label free quantification ratios from welding fume exposure—after exposure/before exposure (NL2/NL1) and day after exposure/before exposure (NL3/NL1). The proteins in this list were detected in more than half of the samples and had a ratio more than 1.3 or less than 0.8. ^a^Not detected in NL1. ^b^Not detected in NL2 and/or NL3.

**Additional file 2.** Canonical pathway results of pooled samples. IPA results of pathways found to be significantly induced when proteins that had a ratio ≥ 1.3 or ≤ 0.8 from additional file 1 were analyzed. After exposure = NL2/NL1. Day after exposure = NL3/NL1.

**Additional file 3.** Upstream analysis for observation immediately after exposure and the day after exposure.

**Additional file 4.** Flowchart for protein list generation and analyses. A total of 336 proteins were detected with the shotgun analysis. Cutoff criteria were used to minimize labor and the time-consuming analysis of redundant proteins. Mechanism hypotheses were generated with the help of pathway analysis and literature findings.

**Additional file 5.** Proteins to be targeted with a SRM method. Proteins with SRM assays from a previously developed method and proteins to be added to the SRM method.

**Additional file 6.** Final SRM method for this study. The final SRM method contained 256 precursors with 811 transitions targeted 130 proteins and 4 in vivo peptide degradation products.

**Additional file 7.** SRM assay development for absolute quantification. Transitions and CE for the light and heavy peptides.

**Additional file 8.** Calibration curves. Calibration curves prepares in nasal lavage matrix: Two sample preparations of the standard curve were made and run during the beginning and the end of the whole runs. They were each injected twice. Calibration curves prepared in MilliQ water matrix: One sample preparation of the standard curve was prepared in MilliQ water as a matrix instead of nasal lavage to confirm that the matrix of the sample was not affecting the quantification of the peptide.

**Additional file 9.** Reproducibility and absolute quantification.

**Additional file 10.** Medical examination of the welders. Medical examination of the welders with lower airway symptoms during the previous month, including skin prick test positivity for a standard panel of aeroallergens, methacholine test (MCH) positivity, and lung function (FVC and FEV_1_ as % of predicted) at the baseline examination before the study. MMP9 concentration (fmol/µL) and lung function (FVC and FEV_1_ as % of predicted) before and after exposure to welding fume particles (A) or filtered air (B). N/A: no data could be obtained due to missing samples.

**Additional file 11.** The significantly changed proteins found with the Wilcoxon signed rank test showed that these canonical pathways were activated due to welding fume exposure. Positive z-score: the canonical pathway was predicted to be activated. Negative z-score: the canonical pathway was predicted to be deactivated.

## Abbreviations

ACN: acetonitrile
MS: mass spectrometry
FA: formic acid
LOD: limit of detection
FDR: false discovery rate
SRM: selected reaction monitoring
IL: interleukin
LC: liquid chromatography
IPA: ingenuity pathway analysis
BCA: bicinchoninic acid
DTT: dithiothreitol
FVC: forced vital capacity
FEV: forced expiratory volume
PD_15_: provocative dose to produce a 15% fall in FEV_1_
MCH: methacholine
MMP9: metalloproteinase 9
A1AT: alpha-1-antitrypsine
A2MG: alpha-2-macrogloboline
APOB: apolipoprotein-B
CV: coefficient of variation
LMM: linear mixed model
ECM: extracellular matrix
LXR/RXR: liver X receptor/retinoid X receptor
