# Supplementary material for: Comprehensive proteome analysis of nasal lavage samples after controlled exposure to welding nanoparticles shows an induced acute phase and a nuclear receptor, LXR/RXR, activation that influence the status of the extracellular matrix
Source: Clin Proteomics. 2018 May 11;15:20. doi: 10.1186/s12014-018-9196-y (PMC5946400; doi:10.1186/s12014-018-9196-y)
Supplement: Supplementary file 1 — Additional file 1. Protein identification and mechanism hypothesis generation from pooled samples. The protein list generated from the analysis of pooled samples. Calculated label free quantification ratios from welding fume exposure—after exposure/before exposure (NL2/NL1) and day after exposure/before exposure (NL3/NL1). The proteins in this list were detected in more than half of the samples and had a ratio more than 1.3 or less than 0.8. aNot detected in NL1. bNot detected in NL2 and/or NL3. [file 12014_2018_9196_MOESM1_ESM.pdf]

Additional file 1

Protein identification and mechanism hypothesis generation from pooled samples:
The protein list generated from the analysis of pooled samples. Calculated label free quantification ratios from welding fume exposure – after exposure/before exposure (NL2/NL1) and day after exposure/before exposure (NL3/NL1). The proteins in this list were detected in more than half of the samples and had a ratio more than 1.3 or less than 0.8. [a] Not detected in NL1. [b] Not detected in NL2 and/or NL3.

AC	Protein name	NL2/NL1	NL3/NL1
P31947	14-3-3 protein sigma	0.77	0.60
P63104	14-3-3 protein zeta/delta	0.77	1.15
Q01518	Adenylyl cyclase-associated protein 1	[a]	[a]
P02763	Alpha-1-acid glycoprotein 1	0.64	0.73
P19652	Alpha-1-acid glycoprotein 2	[a]	[a,b]
P01011	Alpha-1-antichymotrypsin	1.61	2.14
P01009	Alpha-1-antitrypsin	1.00	0.57
P04217	Alpha-1B-glycoprotein	0.79	0.90
P02765	Alpha-2-HS-glycoprotein	0.98	0.68
P01023	Alpha-2-macroglobulin	0.84	0.75
P04083	Annexin A1	0.62	0.89
P12429	Annexin A3	0.76	0.83
P03973	Antileukoproteinase	1.20	0.63
P01008	Antithrombin-III	[a,b]	[a,b]
P02652	Apolipoprotein A-II	1.53	1.03
P06727	Apolipoprotein A-IV	[a]	[a]
Q04118	Basic salivary proline-rich protein 3	1.36	0.99
P02749	Beta-2-glycoprotein 1	0.79	0.49
P61769	Beta-2-microglobulin	1.58	1.16
Q8TDL5	BPI fold-containing family B member 1	1.21	2.13
Q8N4F0	BPI fold-containing family B member 2	0.78	1.02
Q13938	Calcyphosin	0.12	0.14
P62158	Calmodulin	0.67	0.74
Q9NZT1	Calmodulin-like protein 5	0.81	0.70
P31944	Caspase-14	0.88	0.55
P04040	Catalase	[b]	1.42
P49913	Cathelicidin antimicrobial peptide	1.01	1.41
P08311	Cathepsin G	0.73	1.20
P10909	Clusterin	0.98	1.37
Q14019	Coactosin-like protein	[a]	[a]
P23528	Cofilin-1	0.64	0.72
P00751	Complement factor B	0.91	0.74
P08603	Complement factor H	0.79	0.66
P35321	Cornifin-A	1.76	0.81

P04080	Cystatin-B	0.77	0.58
P01034	Cystatin-C	0.63	0.73
P01036	Cystatin-S	0.54	2.73
P54108	Cysteine-rich secretory protein 3	1.27	0.68
P81605	Dermcidin	2.75	0.92
P19957	Elafin	2.30	0.63
P61916	Epididymal secretory protein E1	1.66	1.01
Q9GZZ8	Extracellular glycoprotein lacritin	0.88	2.75
P15311	Ezrin	0.44	0.52
Q01469	Fatty acid-binding protein, epidermal	1.01	0.61
P02675	Fibrinogen beta chain	0.67	0.82
P02679	Fibrinogen gamma chain	0.65	0.74
P20930	Filaggrin	0.96	0.41
P04075	Fructose-bisphosphate aldolase A	0.79	1.11
Q08380	Galectin-3-binding protein	1.01	1.52
O75223	Gamma-glutamylcyclotransferase	0.91	[b]
P09211	Glutathione S-transferase P	0.64	0.39
P04406	Glyceraldehyde-3-phosphate dehydrogenase	0.94	1.47
Q8NBJ4	Golgi membrane protein 1	0.84	0.78
P0DMV9	Heat shock 70 kDa protein 1B	0.70	1.27
P17066	Heat shock 70 kDa protein 6	[b]	0.99
P04792	Heat shock protein beta-1	1.10	0.69
P69905	Hemoglobin subunit alpha	0.38	0.94
P68871	Hemoglobin subunit beta	0.42	0.87
P02790	Hemopexin	1.02	0.66
P16403	Histone H1.2	0.45	0.54
P16401	Histone H1.5	[a,b]	[a,b]
Q99878	Histone H2A type 1-J	[a]	[a]
Q99880	Histone H2B type 1-L	[b]	2.50
P62805	Histone H4	0.94	2.74
P01877	Ig alpha-2 chain C region	1.03	0.54
P01857	Ig gamma-1 chain C region	0.75	0.81
P01859	Ig gamma-2 chain C region	0.51	0.36
P01860	Ig gamma-3 chain C region	0.55	0.63
P01861	Ig gamma-4 chain C region	0.81	0.65
P01766	Ig heavy chain V-III region BRO	[b]	2.37
P01765	Ig heavy chain V-III region TIL	1.06	1.58
P01834	Ig kappa chain C region	1.31	1.48
P04433	Ig kappa chain V-III region VG	1.05	[b]
P01700	Ig lambda chain V-I region HA	2.07	1.84
P80748	Ig lambda chain V-III region LOI	0.91	1.45
P0CG06	Ig lambda-3 chain C regions	3.56	0.63
P01591	Immunoglobulin J chain	1.32	0.97
B9A064	Immunoglobulin lambda-like polypeptide 5	1.10	1.52
P01042	Kininogen-1	[a]	[a,b]

P02788	Lactotransferrin	1.02	0.72
P31025	Lipocalin-1	1.56	2.34
Q6UWW0	Lipocalin-15	1.91	2.78
O75556	Mammaglobin-B	0.77	2.17
P14780	Matrix metalloproteinase-9	0.63	0.82
Q13421	Mesothelin	0.76	0.95
P26038	Moesin	0.56	0.62
P98088	Mucin-5AC	2.39	6.35
Q9HC84	Mucin-5B	1.35	2.81
P24158	Myeloblastin	[a,b]	[a,b]
P05164	Myeloperoxidase	0.68	0.78
P35579	Myosin-9	0.61	0.84
P08246	Neutrophil elastase	0.00	1.33
P80188	Neutrophil gelatinase-associated lipocalin	0.79	0.60
P10153	Non-secretory ribonuclease	[a]	[a,b]
Q02818	Nucleobindin-1	1.49	2.11
P80303	Nucleobindin-2	1.17	1.37
O75594	Peptidoglycan recognition protein 1	[a]	[a,b]
P62937	Peptidyl-prolyl cis-trans isomerase A	0.70	0.94
P23284	Peptidyl-prolyl cis-trans isomerase B	1.03	[b]
P00558	Phosphoglycerate kinase 1	[a]	[a]
P36955	Pigment epithelium-derived factor	0.97	0.76
P05155	Plasma protease C1 inhibitor	[a]	[a]
P13796	Plastin-2	0.63	0.90
P07737	Profilin-1	0.67	0.89
Q99935	Proline-rich protein 1	1.28	8.19
P07602	Prosaposin	1.07	0.67
P02760	Protein AMBP	[a]	[a,b]
P31949	Protein S100-A11	1.47	1.25
P06703	Protein S100-A6	[a,b]	[a]
P31151	Protein S100-A7	3.16	0.84
P05109	Protein S100-A8	0.78	2.56
P06702	Protein S100-A9	1.03	2.28
P00734	Prothrombin	0.82	0.76
P06454	Prothymosin alpha	1.08	[b]
P14618	Pyruvate kinase PKM	[a]	[a,b]
P07998	Ribonuclease pancreatic	2.72	1.36
P0DMR2	Secretoglobin family 1C member 1	2.05	1.55
O95968	Secretoglobin family 1D member 1	[a,b]	[a]
P02787	Serotransferrin	0.87	0.70
P02768-1	Serum albumin	0.95	0.69
P02814	Submaxillary gland androgen-regulated protein 3B	[a]	[a]
P00441	Superoxide dismutase [Cu-Zn]	0.89	[b]
Q6UWP8	Suprabasin	1.28	0.60

P10599	Thioredoxin	0.72	0.79
P37837	Transaldolase	[a,b]	[a,b]
P20061	Transcobalamin-1	1.38	1.39
P29401	Transketolase	0.70	1.17
P02766	Transthyretin	[a]	[a]
Q07654	Trefoil factor 3	1.70	1.15
P06753	Tropomyosin alpha-3 chain	0.79	[b]
Q6P5S2	UPF0762 protein C6orf58	0.76	0.89
P11684	Uteroglobin	0.64	1.03
Q6UXB2	VEGF coregulated chemokine 1	[a]	[a]
P08670	Vimentin	0.67	0.74
P02774	Vitamin D-binding protein	1.00	0.66
Q14508	WAP four-disulfide core domain protein 2	1.31	0.41
Q96DA0	Zymogen granule protein 16 homolog B	1.31	2.59
