# Supplementary material for: Comprehensive proteome analysis of nasal lavage samples after controlled exposure to welding nanoparticles shows an induced acute phase and a nuclear receptor, LXR/RXR, activation that influence the status of the extracellular matrix
Source: Clin Proteomics. 2018 May 11;15:20. doi: 10.1186/s12014-018-9196-y (PMC5946400; doi:10.1186/s12014-018-9196-y)
Supplement: Supplementary file 2 — Additional file 2. Canonical pathway results of pooled samples. IPA results of pathways found to be significantly induced when proteins that had a ratio ≥ 1.3 or ≤ 0.8 from additional file 1 were analyzed. After exposure = NL2/NL1. Day after exposure = NL3/NL1. [file 12014_2018_9196_MOESM2_ESM.pdf]

Additional file 2

Canonical pathway results of pooled samples.

IPA results of pathways found to be significantly induced when proteins that had a ratio ≥1.3 or ≤0.8 from additional file 1 were analyzed. After exposure = NL2/NL1. Day after exposure = NL3/NL1.

Observation time	Canonical pathway	Proteins involved in the pathway	Activity of pathway
After exposure	LXR/RXR Activation	KNG1, TTR, APOA4, ORM1, APOH, APOA2, AMBP, ORM2, S100A8, MMP9, A1BG	Increase
	Acute Phase Response Signaling	TTR, SERPING1, ORM1, APOH, APOA2, AMBP, ORM2, FGB, SERPINA3, FGG	Decrease
	Coagulation System	KNG1, SERPINC1, FGB, FGG	Decrease
	Production of Nitric Oxide and Reactive Oxygen Species in Macrophages	MPO, APOA4, ORM1, APOA2, CAT, ORM2, S100A8	Increase
	Actin Cytoskeleton Signaling	KNG1, MYH9, PFN1, CFL1, EZR, MSN	Decrease
	RhoA Signaling	PFN1, CFL1, EZR, MSN	Decrease
	Dendritic Cell Maturation	B2M, IGHG3, IGHG1, IGHG2	Decrease
Day after exposure	Acute Phase Response Signaling	SERPING1, TTR, HPX, APOH, AHSG, SERPINF1, SERPINA3, F2, FGG, ALB, ORM1, TF, CFB, SERPINA1, A2M	Decrease
	LXR/RXR Activation	HPX, ALB, TTR, APOA4, ORM1, APOH, TF, AHSG, SERPINF1, S100A8, SERPINA1, GC, CLU	Decrease
	Coagulation System	SERPINC1, SERPINA1, A2M, F2, FGG	Increase
	Production of Nitric Oxide and Reactive Oxygen Species in Macrophages	ALB, MPO, APOA4, ORM1, CAT, S100A8, SERPINA1, CLU	Decrease
	Actin Cytoskeleton Signaling	CFL1, EZR, F2, MSN	Decrease
	Phospholipase C Signaling	CALML5, IGHG3, IGHG4, IGHG2	Decrease
