# Supplementary material for: Comprehensive proteome analysis of nasal lavage samples after controlled exposure to welding nanoparticles shows an induced acute phase and a nuclear receptor, LXR/RXR, activation that influence the status of the extracellular matrix
Source: Clin Proteomics. 2018 May 11;15:20. doi: 10.1186/s12014-018-9196-y (PMC5946400; doi:10.1186/s12014-018-9196-y)
Supplement: Supplementary file 3 — Additional file 3. Upstream analysis for observation immediately after exposure and the day after exposure. [file 12014_2018_9196_MOESM3_ESM.pdf]

Additional file 3

Upstream analysis for observation immediately after exposure and the day after exposure.

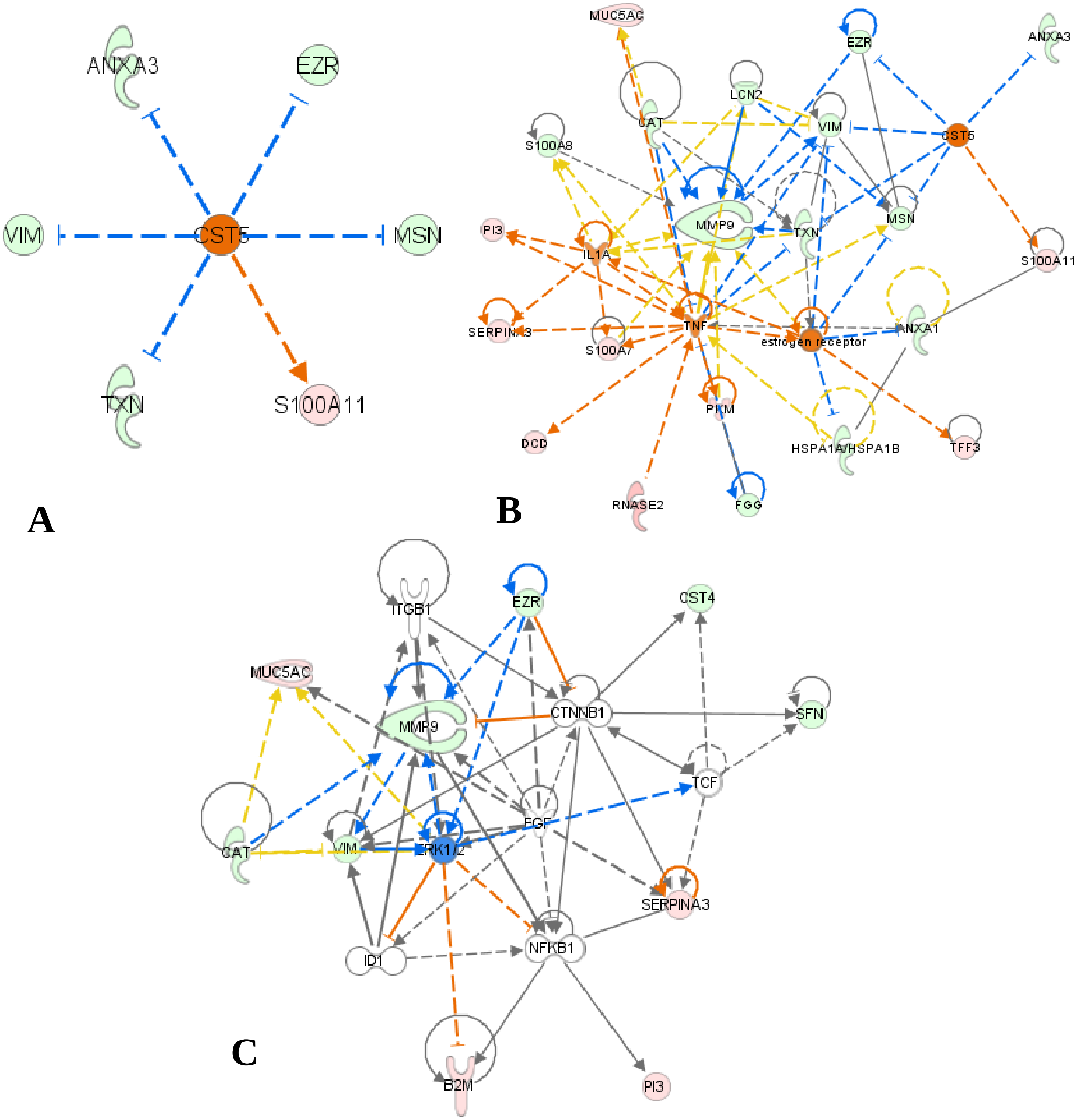

Upstream analysis for observation immediately after exposure; A) CST5 is predicted to be in an activated state. B) Regulators with activation z-scores above 1 were IL1A, CST5, TNF, and estrogen receptor. Canonical pathways connected to these regulators are glucocorticoid receptor signaling, granulocyte adhesion diapedesis, and agranulocyte adhesion diapedesis. C) According to the mechanistic networks, there were 3–7 target molecules downstream from the regulators with 2–4 regulators in the network, CTNNB1, ITGB1, NFKB1, ID1, EGF, ERK 1/2, and TCF. Canonical pathways connected to these regulators were regulation of the epithelial–mesenchymal transition pathway, ILK signaling, and leukocyte extravasation signaling.

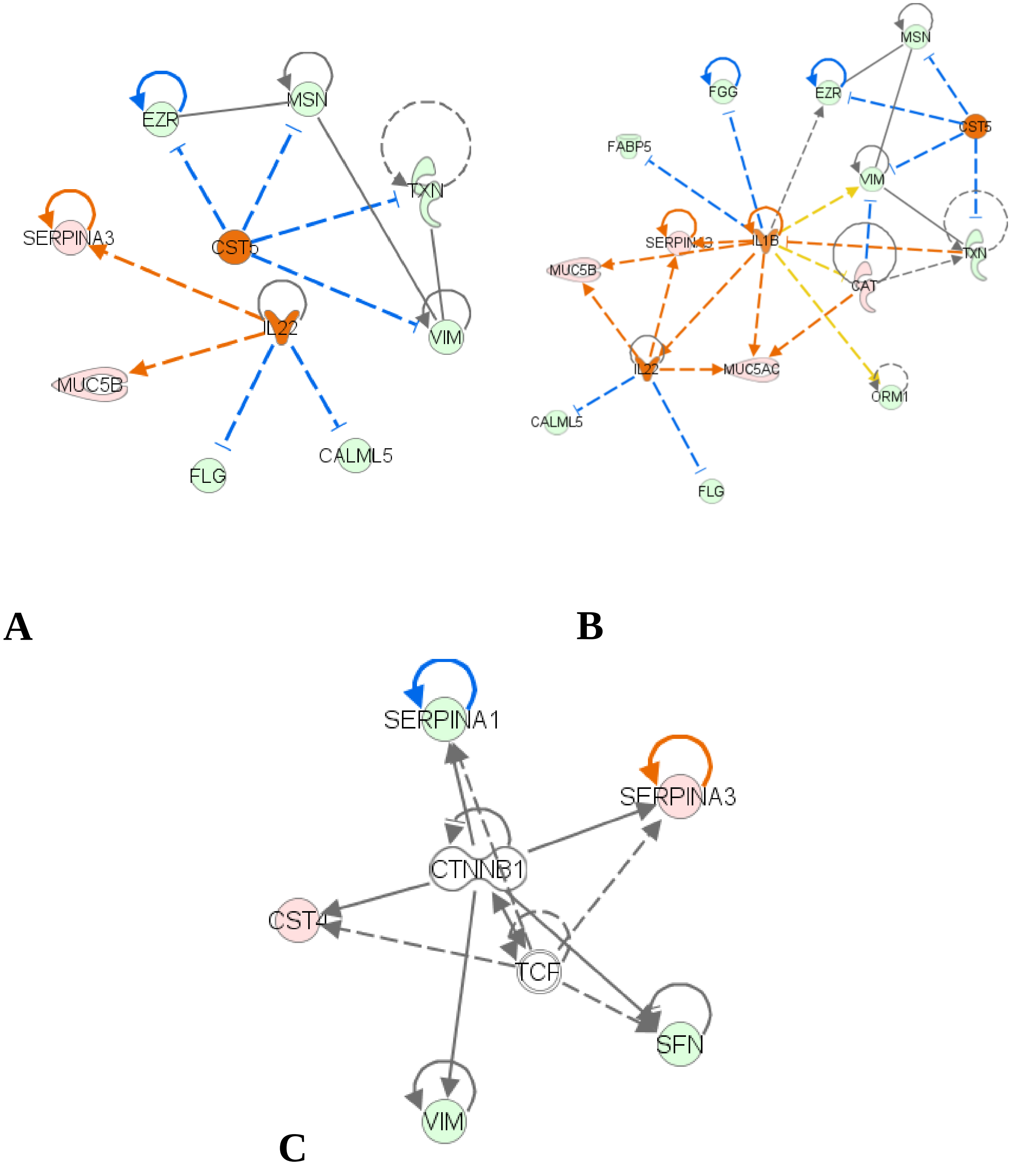

Upstream analysis for observation the day after exposure A) Upstream regulators predicted to be in an activated state were IL22 and CST5. B) Regulators with activation z-scores above 1 were IL1B, CST5, and IL22. Canonical pathways connected to these regulators were acute phase response signaling, signaling by Rho family GTPases, and LPS/IL-1-mediated inhibition of RXR function. C) According to the mechanistic networks, there were five target molecules downstream from the regulators with two regulators in the network, CTNNB1 and TCF. Canonical pathways connected to these were protein kinase A signaling and acute phase response signaling.
