# Supplementary material for: Comprehensive proteome analysis of nasal lavage samples after controlled exposure to welding nanoparticles shows an induced acute phase and a nuclear receptor, LXR/RXR, activation that influence the status of the extracellular matrix
Source: Clin Proteomics. 2018 May 11;15:20. doi: 10.1186/s12014-018-9196-y (PMC5946400; doi:10.1186/s12014-018-9196-y)
Supplement: Supplementary file 4 — Additional file 4. Flowchart for protein list generation and analyses. A total of 336 proteins were detected with the shotgun analysis. Cutoff criteria were used to minimize labor and the time-consuming analysis of redundant proteins. Mechanism hypotheses were generated with the help of pathway analysis and literature findings. [file 12014_2018_9196_MOESM4_ESM.pdf]

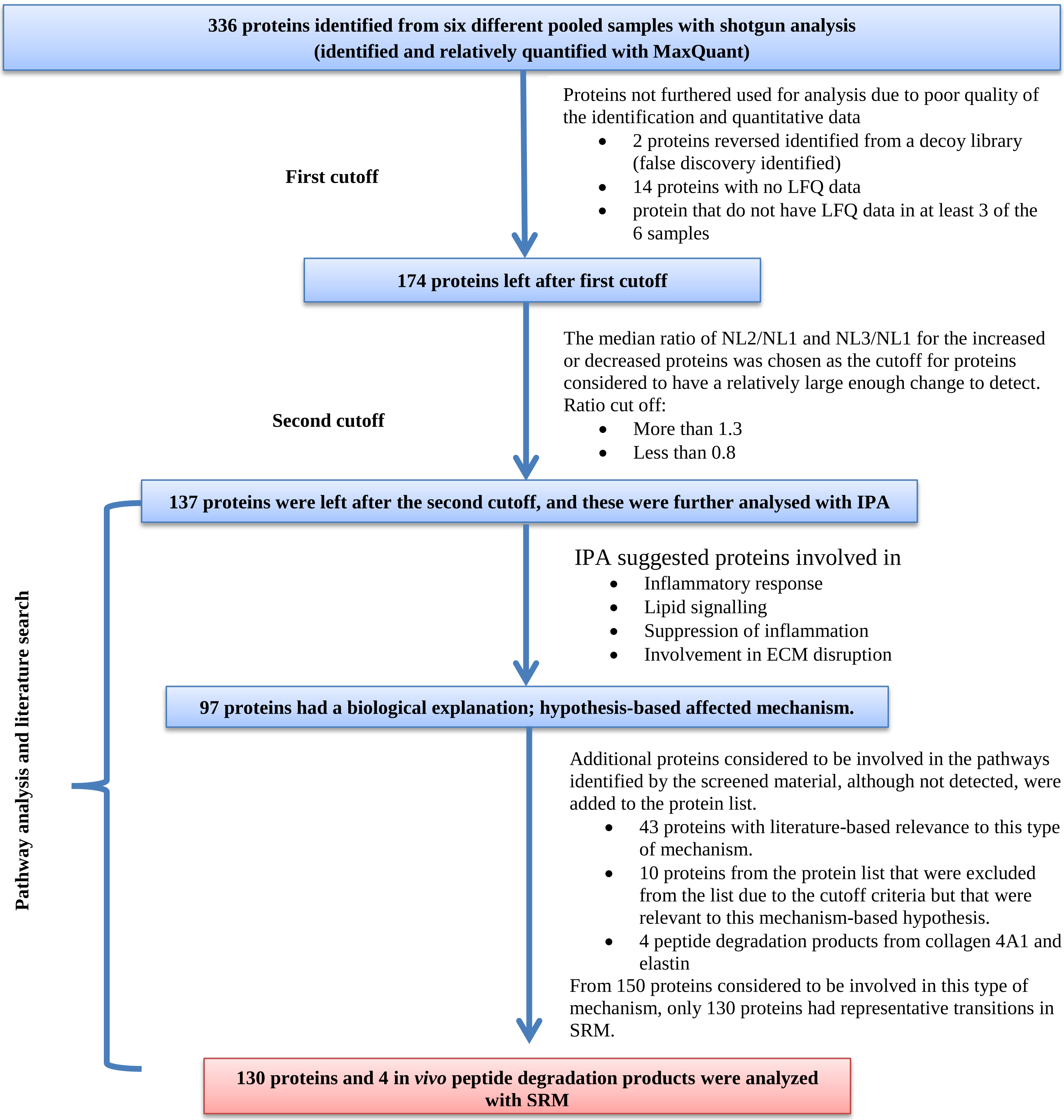

Additional file 4.
Flowchart for protein list generation and analyses.
A total of 336 proteins were detected with the shotgun analysis. Cutoff criteria were used to minimize labor and the time-consuming analysis of redundant proteins. Mechanism hypotheses were generated with the help of pathway analysis and literature findings.
