# Supplementary material for: Comprehensive proteome analysis of nasal lavage samples after controlled exposure to welding nanoparticles shows an induced acute phase and a nuclear receptor, LXR/RXR, activation that influence the status of the extracellular matrix
Source: Clin Proteomics. 2018 May 11;15:20. doi: 10.1186/s12014-018-9196-y (PMC5946400; doi:10.1186/s12014-018-9196-y)
Supplement: Supplementary file 8 — Additional file 8. Calibration curves. Calibration curves prepares in nasal lavage matrix: Two sample preparations of the standard curve were made and run during the beginning and the end of the whole runs. They were each injected twice. Calibration curves prepared in MilliQ water matrix: One sample preparation of the standard curve was prepared in MilliQ water as a matrix instead of nasal lavage to confirm that the matrix of the sample was not affecting the quantification of the peptide. [file 12014_2018_9196_MOESM8_ESM.pdf]

Additional file 8: Calibration curves

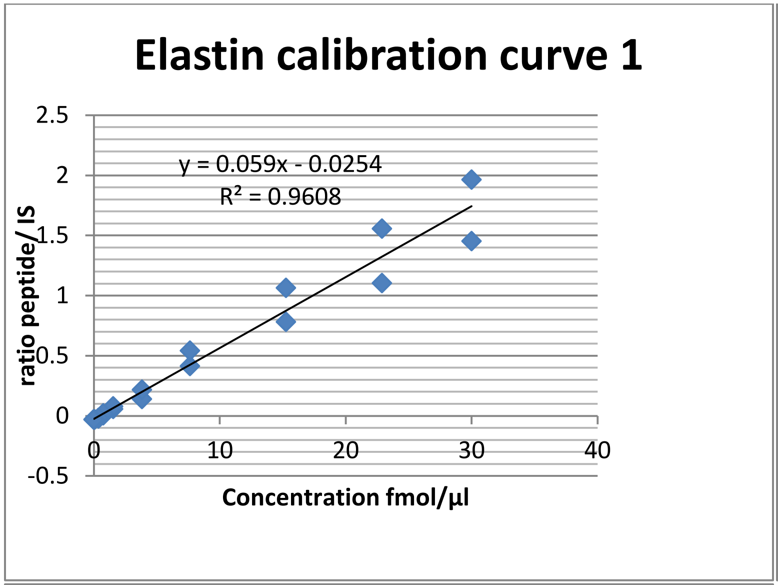

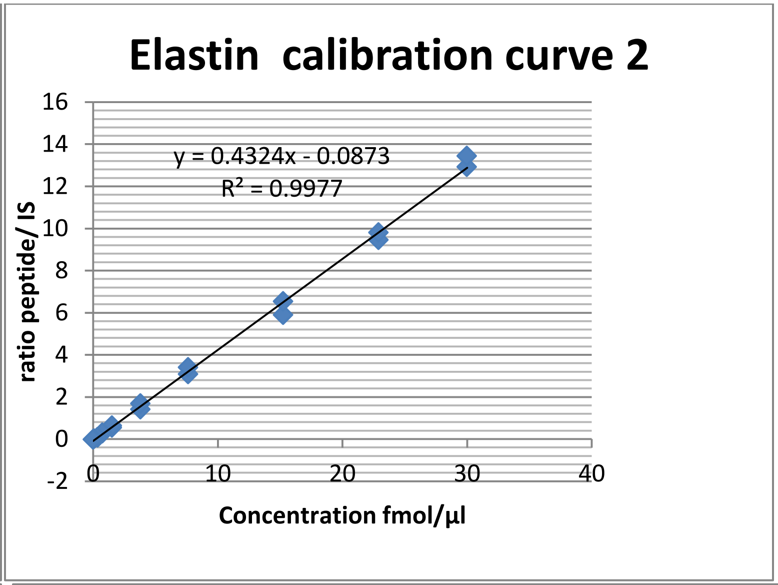

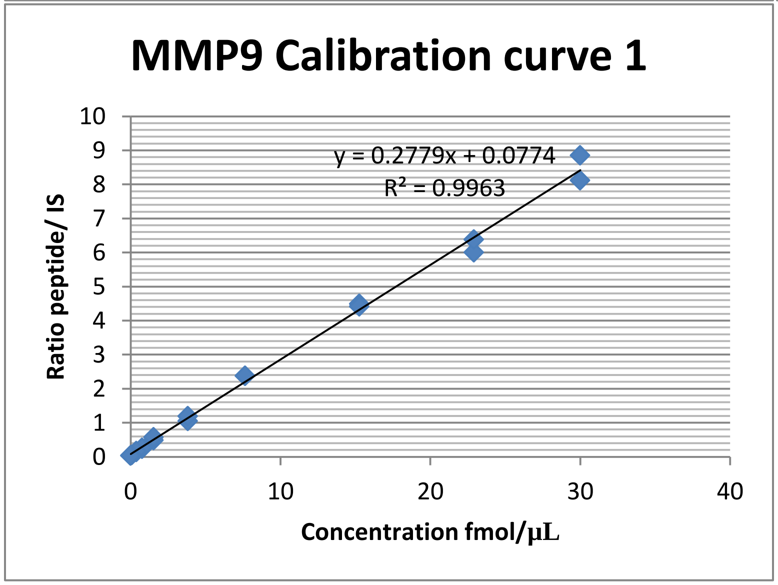

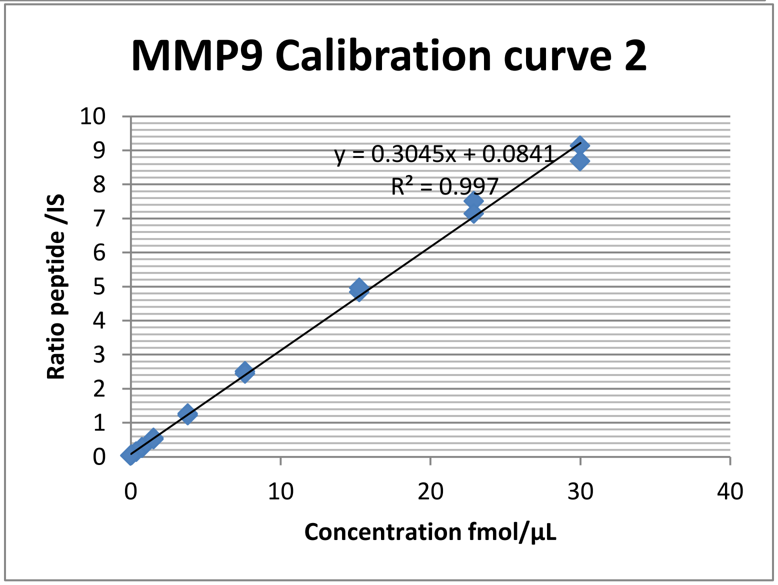

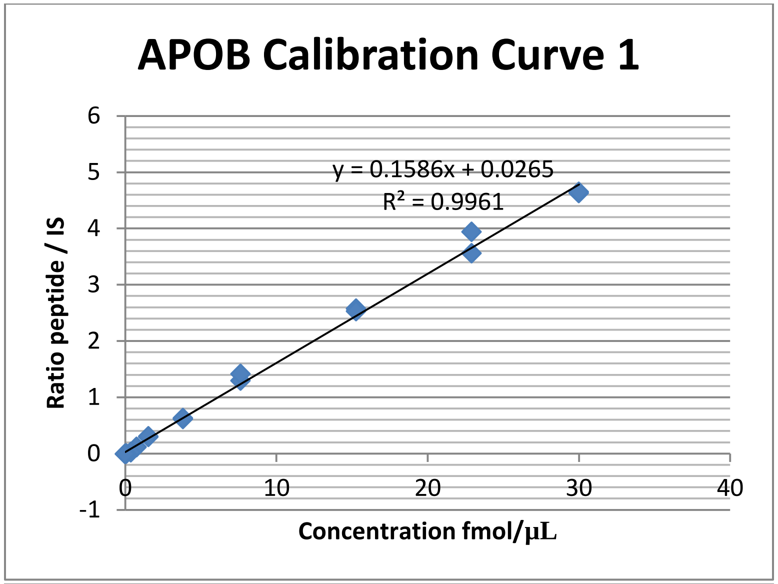
APOB Calibration Curve 1
y = 0.1586x + 0.0265
R² = 0.9961
Ratio peptide / IS
Concentration fmol/µL
6
5
4
3
2
1
0
-1
0
10
20
30
40

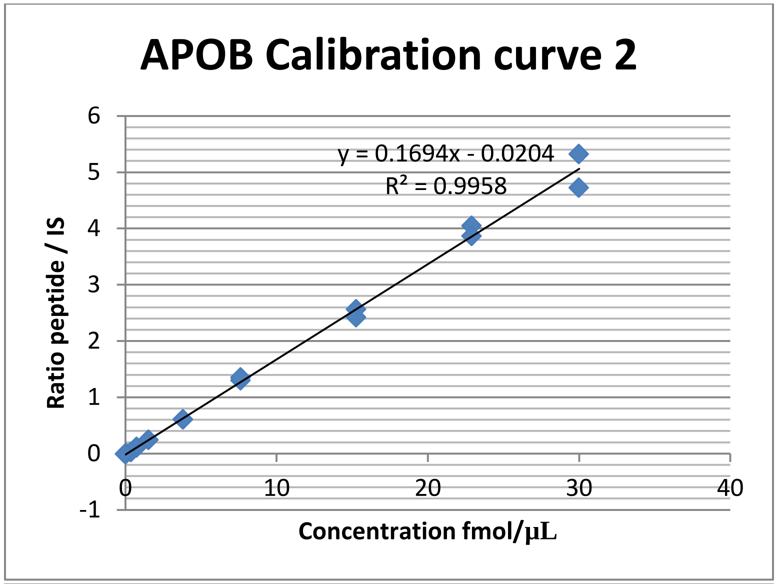
APOB Calibration curve 2
y = 0.1694x - 0.0204
R² = 0.9958
Ratio peptide / IS
Concentration fmol/µL
6
5
4
3
2
1
0
-1
0
10
20
30
40

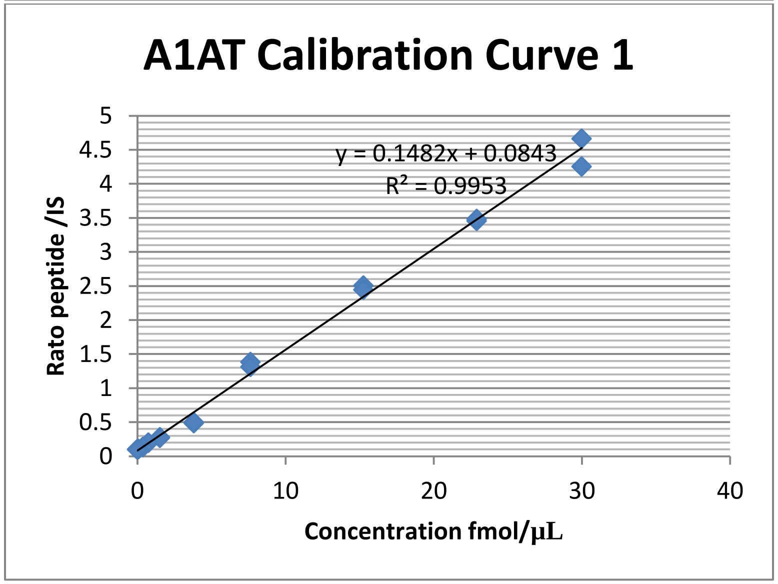
A1AT Calibration Curve 1
y = 0.1482x + 0.0843
R² = 0.9953
Rato peptide /IS
Concentration fmol/µL
5
4.5
4
3.5
3
2.5
2
1.5
1
0.5
0
0
10
20
30
40

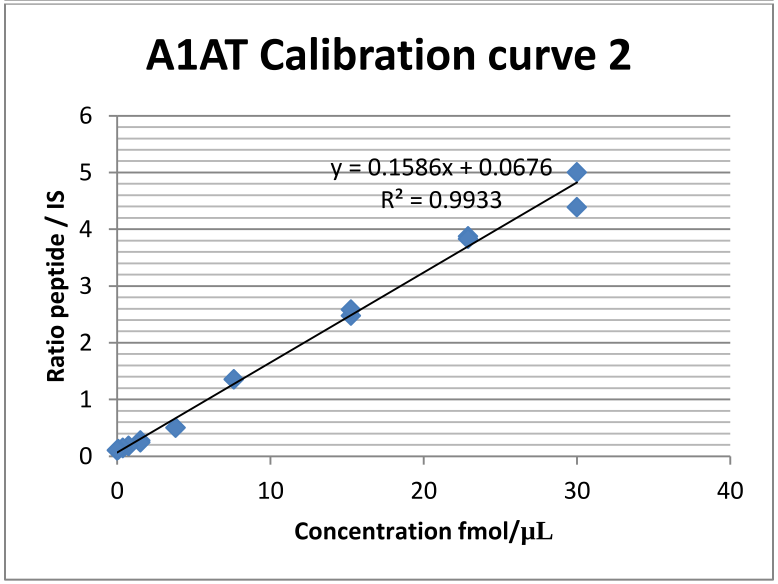
A1AT Calibration curve 2
y = 0.1586x + 0.0676
R² = 0.9933
Ratio peptide / IS
Concentration fmol/µL
6
5
4
3
2
1
0
0
10
20
30
40

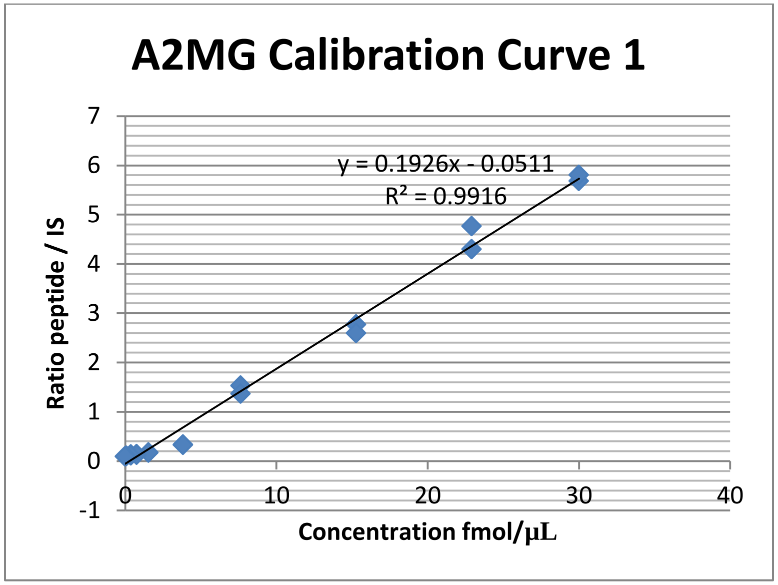

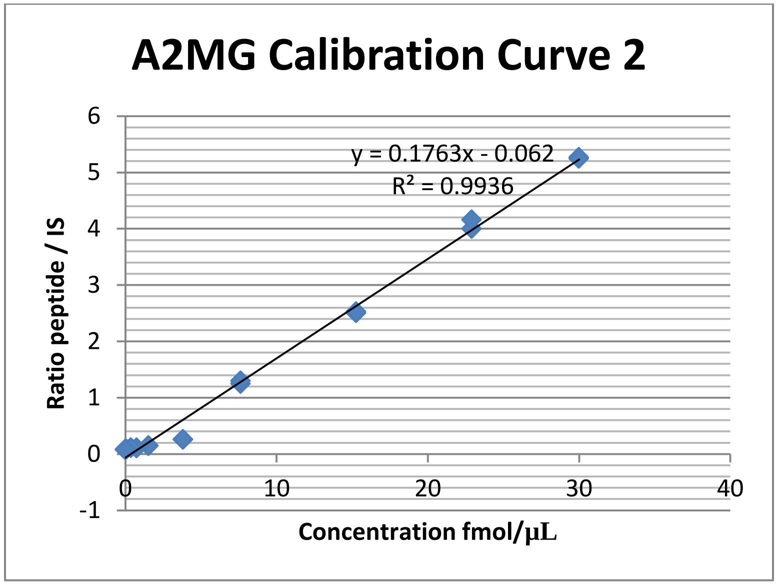

Calibration curves. Two sample preparations of the standard curve were made and run during the beginning and the end of the whole runs. They were each injected twice. These were prepared in a matrix of pooled nasal lavage.

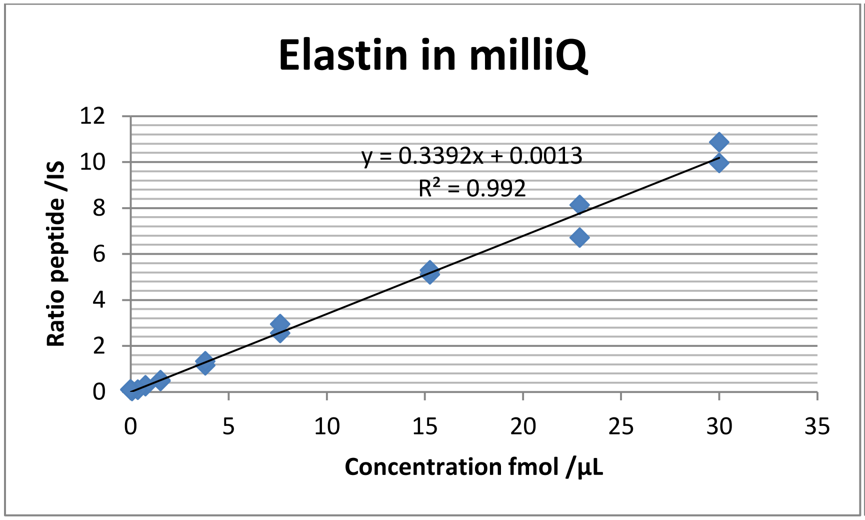
Elastin in milliQ
y = 0.3392x + 0.0013
R² = 0.992
Ratio peptide /IS
Concentration fmol /µL
0 2 4 6 8 10 12
0 5 10 15 20 25 30 35

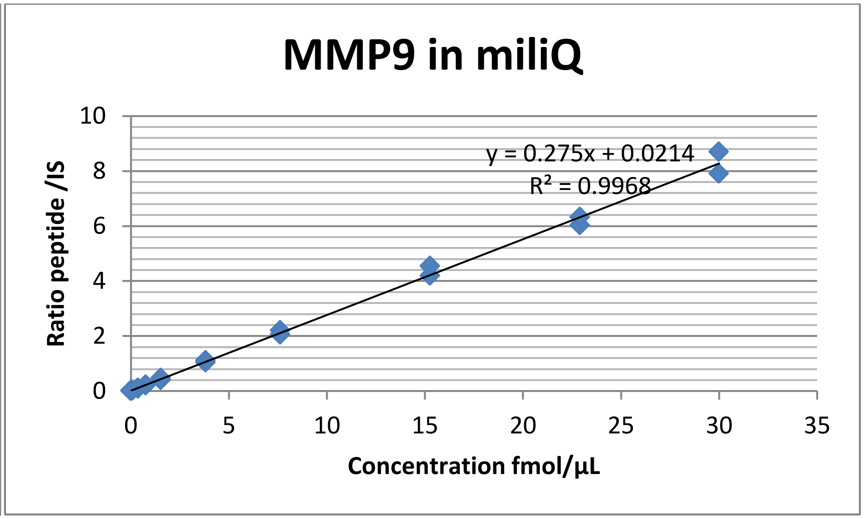
MMP9 in miliQ
y = 0.275x + 0.0214
R² = 0.9968
Ratio peptide /IS
Concentration fmol/µL
0 2 4 6 8 10
0 5 10 15 20 25 30 35

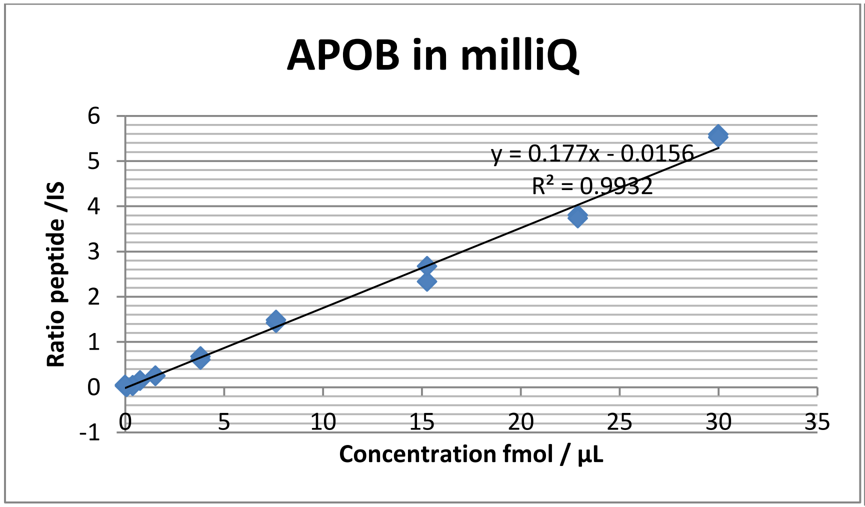
APOB in milliQ
y = 0.177x - 0.0156
R² = 0.9932
Ratio peptide /IS
Concentration fmol / µL
-1 0 1 2 3 4 5 6
0 5 10 15 20 25 30 35

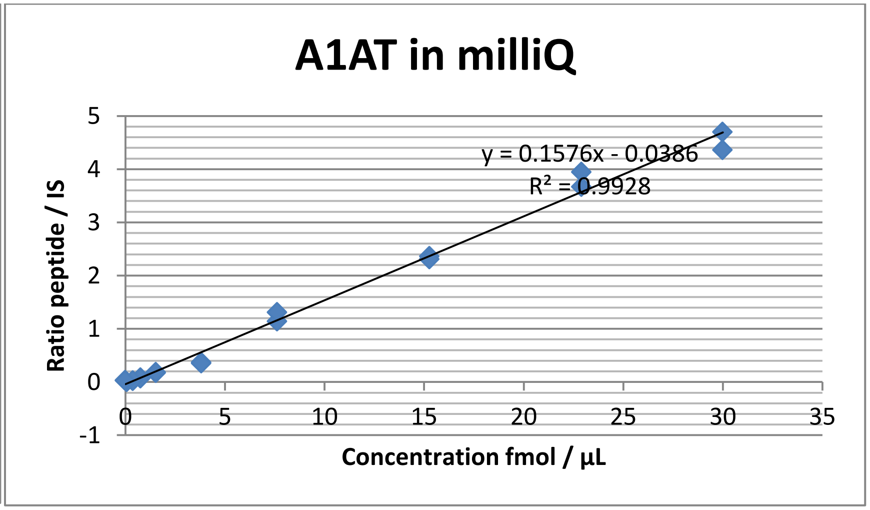
A1AT in milliQ
y = 0.1576x - 0.0386
R² = 0.9928
Ratio peptide / IS
Concentration fmol / µL
-1 0 1 2 3 4 5
0 5 10 15 20 25 30 35

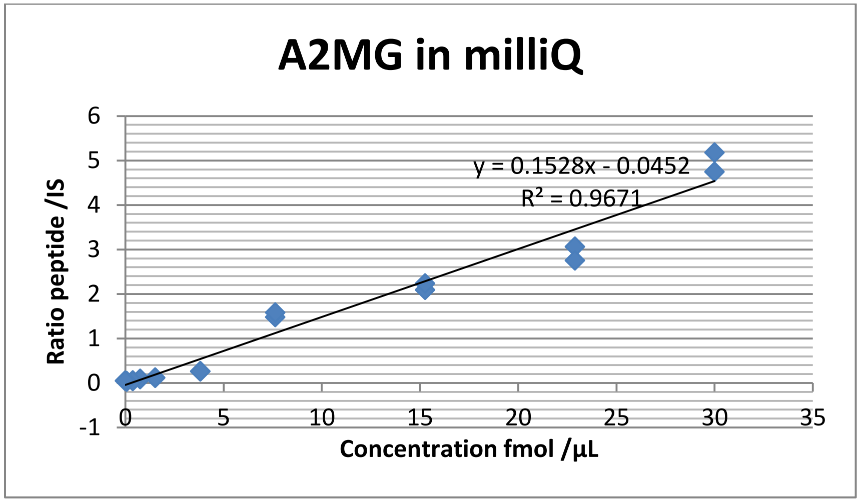

Calibration curves. One sample preparation of the standard curve was prepared in MilliQ water as a matrix instead of nasal lavage to confirm that the matrix of the sample was not affecting the quantification of the peptide.
