# Supplementary material for: Comprehensive proteome analysis of nasal lavage samples after controlled exposure to welding nanoparticles shows an induced acute phase and a nuclear receptor, LXR/RXR, activation that influence the status of the extracellular matrix
Source: Clin Proteomics. 2018 May 11;15:20. doi: 10.1186/s12014-018-9196-y (PMC5946400; doi:10.1186/s12014-018-9196-y)
Supplement: Supplementary file 9 — Additional file 9. Reproducibility and absolute quantification. [file 12014_2018_9196_MOESM9_ESM.pdf]

Additional file 9

Reproducibility and absolute quantification.

Reproducibility was calculated from pooled quality control samples (nasal lavage sample spiked with isotopically labeled peptides). There was little variation between days when assessing all isotopically labeled peptides except elastin. The isotopically labeled peptide for elastin did not show the same total peak area variation as the endogenous peptide, thus this peptide could not be used for quantification. Also, a large discrepancy between the slopes of the calibration curves prepared in water compared to nasal lavage matrix was observed (Additional file 8). Elastin was therefore not quantified.

The APOB endogenous peptide had a very low signal to noise ratio. Therefore, the isotopically labeled peptide of APOB was difficult to evaluate. Most of the samples had levels similar to, or lower than, the LOD for APOB.
