# Supplementary material for: Comprehensive proteome analysis of nasal lavage samples after controlled exposure to welding nanoparticles shows an induced acute phase and a nuclear receptor, LXR/RXR, activation that influence the status of the extracellular matrix
Source: Clin Proteomics. 2018 May 11;15:20. doi: 10.1186/s12014-018-9196-y (PMC5946400; doi:10.1186/s12014-018-9196-y)
Supplement: Supplementary file 10 — Additional file 10. Medical examination of the welders. Medical examination of the welders with lower airway symptoms during the previous month, including skin prick test positivity for a standard panel of aeroallergens, methacholine test (MCH) positivity, and lung function (FVC and FEV1 as % of predicted) at the baseline examination before the study. MMP9 concentration (fmol/µL) and lung function (FVC and FEV1 as % of predicted) before and after exposure to welding fume particles (A) or filtered air (B). N/A: no data could be obtained due to missing samples. [file 12014_2018_9196_MOESM10_ESM.pdf]

Additional file 10

Medical examination of the welders.

Medical examination of the welders with lower airway symptoms during the previous month, including skin prick test positivity for a standard panel of aeroallergens, methacholine test (MCH) positivity, and lung function (FVC and FEV_1 as % of predicted) at the baseline examination before the study. MMP9 concentration (fmol/µL) and lung function (FVC and FEV_1 as % of predicted) before and after exposure to welding fume particles (A) or filtered air (B). N/A: no data could be obtained due to missing samples.

	Baseline medical examination					Exposure	MMP9 concentration		FVC %		FEV1 %	
	Welding years	Skin-prick test	MCH PD_{15}	FVC%	FEV_1 %		Before	After	Before	After	Before	After
1	26			114	114	A	0.41	0.37	119	115	116	113
						B	0.42	0.52	121	122	126	120
2	22	positive		93	101	A	0.61	0.11	88	87	97	93
						B	0.02	0.03	91	90	102	103
3	34			101	100	A	0.12	0.1	98	105	100	107
						B	0.05	0.1	111	104	109	101
4	37			72	74	A	0.11	0.11	66	70	75	78
						B	0.12	0.15	70	73	76	81
5	9		positive	79	71	A	0.02	0.02	79	87	72	77
						B	0.16	0.12	80	81	70	71
6	13			105	108	A	0.16	0.08	106	108	108	110
						B	0.15	0.11	105	103	106	105
7	2	positive		99	101	A	0.56	0.16	103	100	104	102
						B	0.22	0.11	101	100	102	99
8	11	positive	positive	82	66	A	N/A	0.05	74	78	60	62
						B	0.08	0.08	65	64	54	49
9	4			85	90	A	0.09	0.05	89	90	91	91
						B	0.04	0.03	89	91	91	91
10	14			114	132	A	0.37	0.27	111	119	132	134
						B	0.3	0.29	120	118	136	133
11	39			108	127	A	0.12	0.24	97	97	110	114
						B	0.17	0.22	101	101	118	118
