# Supplementary material for: Comprehensive proteome analysis of nasal lavage samples after controlled exposure to welding nanoparticles shows an induced acute phase and a nuclear receptor, LXR/RXR, activation that influence the status of the extracellular matrix
Source: Clin Proteomics. 2018 May 11;15:20. doi: 10.1186/s12014-018-9196-y (PMC5946400; doi:10.1186/s12014-018-9196-y)
Supplement: Supplementary file 11 — Additional file 11. The significantly changed proteins found with the Wilcoxon signed rank test showed that these canonical pathways were activated due to welding fume exposure. Positive z-score: the canonical pathway was predicted to be activated. Negative z-score: the canonical pathway was predicted to be deactivated. [file 12014_2018_9196_MOESM11_ESM.docx]

# Additional file 11

The significantly changed proteins found with the Wilcoxon signed rank test showed that these canonical pathways were activated due to welding fume exposure. Positive z-score: the canonical pathway was expected to be activated. Negative z-score: the canonical pathway was expected to be deactivated. Zero z-score: the direction of the proteins in the dataset are equally likely to have an activating as an inactivating capacity on the pathway

|  | Pathway | z-score | proteins |
| --- | --- | --- | --- |
| After exposure | Leukocyte Extravasation Signaling | -1 | F11R, MMP9, MSN, EZR |
|  | LXR/RXR Activation | 1.34 | AGT, APOA4, MMP9, AHSG, ALB |
| The day after exposure | Dendritic Cell Maturation | 2 | IL1RN, IGHG2, IGHG1, COL1A2 |
|  | Production of Nitric Oxide and Reactive Oxygen Species in Macrophages | 0 | PON1, APOA4, CAT, MPO, ALB |
|  | Acute Phase Response Signaling | 0.71 | AGT, A2M, TF, FN1, IL1RN, SERPINF1, APOH, HPX, AMBP, ALB |
|  | LXR/RXR Activation | 1.26 | AGT, PON1, APOA4, TF, IL1RN, SERPINF1, APOH, HPX, AMBP, ALB |
